# Ferroptosis of select skin epithelial cells initiates and maintains chronic systemic immune-mediated psoriatic disease

**DOI:** 10.1172/JCI183219

**Published:** 2024-11-21

**Authors:** Kavita Vats, Hua Tian, Kunal Singh, Yulia Y. Tyurina, Louis J. Sparvero, Vladimir A. Tyurin, Oleg Kruglov, Alexander Chang, Jiefei Wang, Felicia Green, Svetlana N. Samovich, Jiying Zhang, Ansuman Chattopadhyay, Natalie Murray, Vrusha K. Shah, Alicia R. Mathers, Uma R. Chandran, Joseph M. Pilewski, John A. Kellum, Sally E. Wenzel, Hülya Bayır, Valerian E. Kagan, Yuri L. Bunimovich

**Affiliations:** 1 Department of Dermatology,; 2 Center for Free Radical and Antioxidant Health, Department of Environmental Health and Occupational Health, and; 3 Department of Biomedical Informatics, University of Pittsburgh, Pittsburgh, Pennsylvania, USA.; 4 Biomolecular Imaging Lab, Rosalind Franklin Institute, Didcot, United Kingdom.; 5 Molecular Biology Information Service, Health Sciences Library System,; 6 School of Medicine,; 7 Department of Immunology,; 8 Department of Medicine, and; 9 Center for Critical Care Nephrology, Department of Critical Care Medicine, University of Pittsburgh, Pittsburgh, Pennsylvania, USA.; 10 Department of Pediatrics, Division of Critical Care and Hospital Medicine, Redox Health Center, Vagelos College of Physicians and Surgeons, Columbia University Irving Medical Center, New York, New York, USA.

**Keywords:** Dermatology, Inflammation, Autoimmune diseases, Cell stress, Skin

## Abstract

Dysregulations of epithelial-immune interactions frequently culminate in chronic inflammatory diseases of the skin, lungs, kidneys, and gastrointestinal tract. Yet, the intraepithelial processes that initiate and perpetuate inflammation in these organs are poorly understood. Here, by utilizing redox lipidomics we identified ferroptosis-associated peroxidation of polyunsaturated phosphatidylethanolamines in the epithelia of patients with asthma, cystic fibrosis, psoriasis, and renal failure. Focusing on psoriasis as a disease model, we used high-resolution mass spectrometry imaging and identified keratin 14–expressing (K14-expressing) keratinocytes executing a ferroptotic death program in human psoriatic skin. Psoriatic phenotype with characteristic Th1/Th17 skin and extracutaneous immune responses was initiated and maintained in a murine model designed to actuate ferroptosis in a fraction of K14^+^ glutathione peroxidase 4–deficient (Gpx4-deficient) epidermal keratinocytes. Importantly, an antiferroptotic agent, liproxstatin-1, was as effective as clinically relevant biological IL-12/IL-23/TNF-α–targeting therapies or the depletion of T cells in completely abrogating molecular, biochemical, and morphological features of psoriasis. As ferroptosis in select epidermal keratinocytes triggers and sustains a pathological psoriatic multiorgan inflammatory circuit, we suggest that strategies targeting ferroptosis or its causes may be effective in preventing or ameliorating a variety of chronic inflammatory diseases.

## Abstract 2

This work describes how ferroptosis, a specific programmed death mechanism, of select skin epithelial cells causes chronic systemic immune-mediated psoriatic disease.

## Graphical abstract

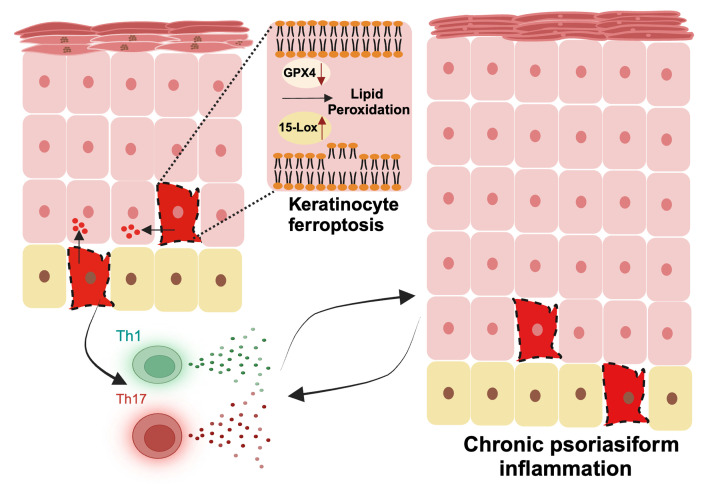

## Introduction

The dysregulation of epithelial-immune crosstalk leads to a multitude of chronic inflammatory diseases affecting respiratory, gastrointestinal, renal, and integumentary systems. However, the mechanisms underlying the initiation and the chronicity of epithelial inflammation are not understood. We have previously reported that ferroptosis-associated redox imbalance in airway and renal epithelium contributes to and reflects disease severity and clinical outcomes (1–3). Yet direct evidence that ferroptosis and associated peroxidation of polyunsaturated phosphatidylethanolamines (PEs) of the epithelial cells may commence and perpetuate a pathogenic inflammatory loop in an organ-specific disease is lacking.

Psoriasis is one of the most prevalent chronic inflammatory diseases involving the dysregulation of the epithelial immune microenvironment and is characterized by hyperproliferation and aberrant differentiation of the skin keratinocytes (KCs) (4, 5). Psoriasis is associated with metabolic syndrome, and individuals living with psoriasis are at an increased risk of developing psoriatic arthritis, colitis, cardiovascular disease, and other comorbidities (4). Genetic, environmental, and immunological factors have been implicated in the pathogenesis of psoriasis, similarly to that of other chronic inflammatory diseases (4, 6). Interactions between the epithelial KCs and the immune cells are thought to mediate the disease by stimulating the production of cytokines, chemokines, and growth factors typically implicated in psoriasis; however, the causes and cell populations involved in triggering and maintaining chronic skin and extracutaneous inflammation have not been established (6, 7). Disrupted lipid metabolism, oxidative stress, and the accumulation of lipid peroxidation products in the skin and blood of psoriasis patients are known features of the disease, but their relevance to psoriasis pathogenesis is also unclear (8–11). Several studies have suggested that the dysregulation of thiol-based redox system and heightened lipid peroxidation, which are hallmarks of ferroptosis, may play an etiological role in psoriasis (11–14).

Here, we used psoriasis as a model to investigate the role of epithelial ferroptosis in chronic systemic immune-mediated diseases. We found that the accumulation of pro-ferroptotic oxidized arachidonic acid–PE (oxPE) species in keratin 14–expressing (K14-expressing) KCs of the epidermis is a prominent feature of psoriasis, and is accompanied by downregulation of glutathione peroxidase 4 (Gpx4), a major regulator of ferroptosis (15), and upregulation of a critical oxPE death signal generator, 15-lipoxygenase-2 (15-LOX-2) (16). Genetic induction of ferroptosis in a small fraction of K14^+^ KCs in mouse epidermis via the deletion of *Gpx4* was sufficient to initiate and maintain a phenotype with cutaneous and systemic inflammation closely resembling psoriasis, and to activate canonical signaling pathways and multiomics biomarkers linked to the disease. The psoriasiform inflammation produced by K14^+^ KC ferroptosis was effectively treated with the pharmacological inhibition of ferroptosis, as well as with depletion of T lymphocytes or current standard-of-care biological anti–IL-12/IL-23/TNF-α therapies. Collectively, our results directly connect the ferroptotic process of select epidermal KCs to psoriasis pathogenesis and illustrate how regulated epithelial cell death by ferroptosis coordinates immune responses during the initiation and the maintenance of a chronic systemic inflammatory disease.

## Results

### In inflammatory diseases of the skin and other organs, epithelial cells undergo lipid peroxidation and ferroptosis.

**Figure 1:**
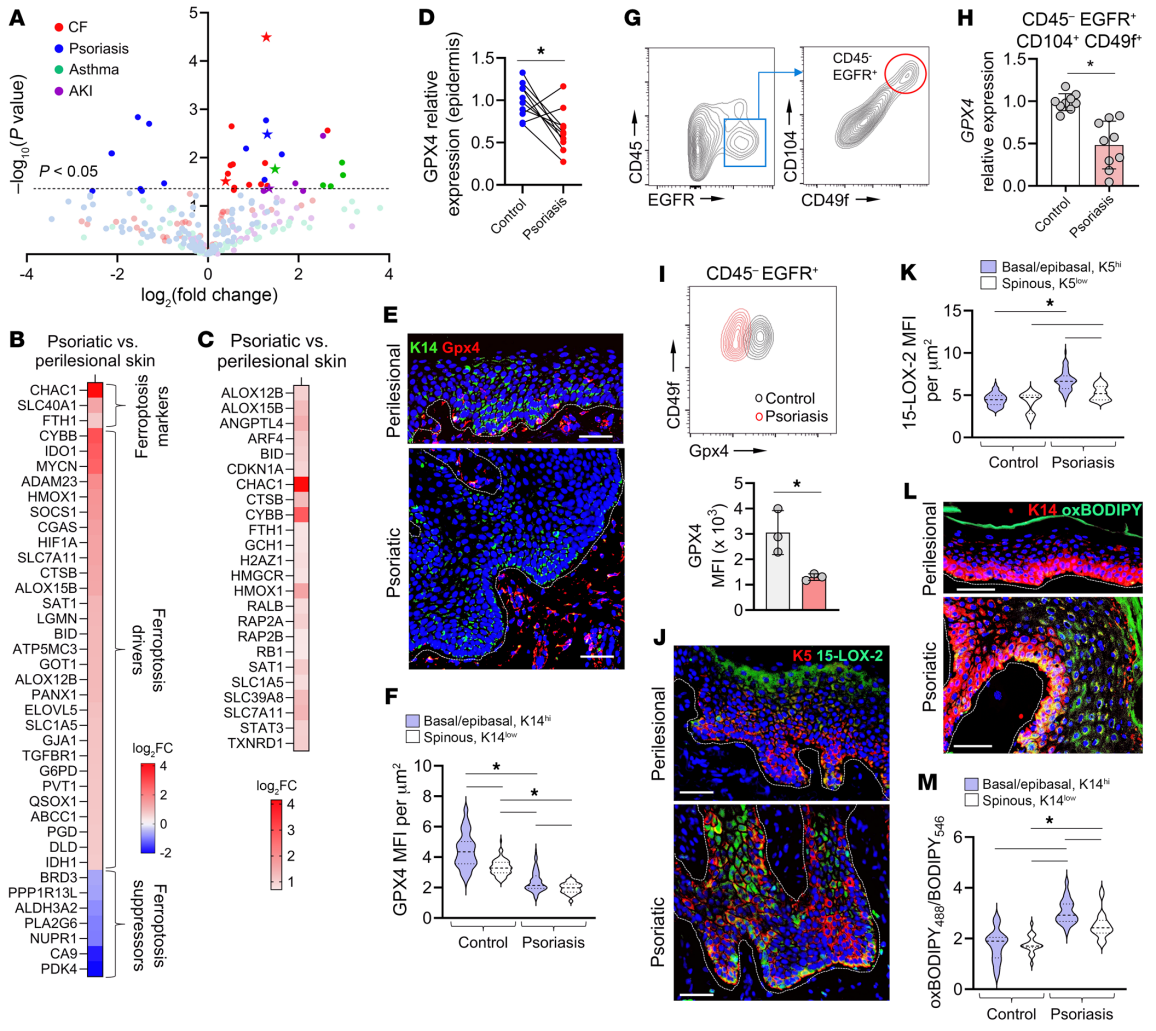
Pro-ferroptotic epidermal oxPE signals in psoriasis are associated with decreased Gpx4 and increased 15-LOX-2 levels. (**A**) Volcano plotof oxidized PE species (with 1–3 [O]) measured by LC-MS from pulmonary airway epithelium of patients with cystic fibrosis (CF) versus non-CF patients (red circles), from patient-matched psoriatic lesional versus control perilesional epidermis (blue circles), from bronchial epithelium of patients with severe asthma versus asthma-free patients (green circles), and from proximal and distal renal tubular epithelium of non-recovered versus recovered patients after acute kidney injury (AKI; purple circles). Ferroptosis-specific oxPE signals [PE(36:4)+2O, PE(36:5)+2O, PE(38:4)+2O] are indicated by stars. (**B** and **C**) Gene expression of ferroptosis markers and regulators in psoriatic versus patient-matched perilesional skin identified by the ferroptosis database FerrDb (**B**) or QIAGEN Ingenuity Pathway Analysis (**C**); *n* = 3. (**D**) Relative expression of *Gpx4* in psoriatic and patient-matched nonlesional epidermis; *n* = 10. (**E** and **F**) Immunofluorescence (IF) images of psoriatic and perilesional skin (**E**) and mean fluorescence intensities (MFIs) per square micrometer of Gpx4 in basal/epibasal and spinous layers (**F**); *n* = 80 regions of interest (ROIs) per group, 3 patients. (**G**) Isolation of CD45^–^EGFR^+^CD104^+^CD49f^+^ basal KCs from human epidermis by FACS. (**H**) Relative Gpx4 expression in basal KCs isolated from psoriatic or control (nonlesional) epidermis as shown in **G**; *n* = 9. (**I**) Flow cytometry (top) and MFI (bottom) of Gpx4 in basal KCs from psoriatic versus control skin; *n* = 3. (**J** and **K**) IF images of psoriatic and perilesional skin (**J**) and 15-LOX-2 MFI per square micrometer in basal/epibasal and spinous layers (**K**); *n* = 54 ROIs per group, 3 patients. (**L** and **M**) IF images of psoriatic and perilesional skin (**L**) and oxBODIPY_488_/BODIPY_546_ (MFI/MFI) in basal/epibasal and spinous KCs (**M**); *n* = 21 ROIs per group, 3 patients. Dermal-epidermal junction is outlined in all IF images by white dashed lines. Data are means ± SD. Two-tailed Student’s *t* test (**D**, **H**, and **I**), 1-way ANOVA (**F**, **K**, and **M**); *P < 0.05. Scale bars: 50 μm.

To comprehensively profile oxidative lipidomics of several human inflammatory diseases involving epithelial tissues, we performed liquid chromatography–mass spectrometry (LC-MS) analyses of pulmonary airway epithelium obtained from patients with asthma and cystic fibrosis, urine cell pellets obtained from patients with acute kidney injury, and skin obtained from patients with psoriasis (Supplemental Figure 1, A–E, and Supplemental Table 1; supplemental material available online with this article; https://doi.org/10.1172/JCI183219DS1) (1–3). In all of these diseases we found significant increases in the levels of oxidized phospholipids (oxPLs), particularly ferroptosis-specific peroxidized oxPE death signals such as PE(36:4)+2O and PE(38:4)+2O, which were among the most abundant oxPL species in the clinical samples (Figure 1A). As the epithelial cells made up the majority of the analyzed bronchial, urine, and skin samples, we reasoned that ferroptosis of the epithelial cells may drive an inflammatory response in these conditions. To test this, we further focused on psoriasis as a model chronic inflammatory disease affecting the epithelium, with localized manifestation in the skin as well as systemic involvement of other organs.

While it was previously suggested that the ferroptotic process may occur in the KCs of human psoriatic skin (12), direct evidence of KC ferroptosis in psoriasis up to now has not been obtained. Cultured KCs are susceptible to RSL3- or erastin-induced ferroptosis and are protected by the ferroptosis inhibitors ferrostatin-1 (Fer-1), liproxstatin-1 (Lip-1), and baicalein (Supplemental Figure 2A). To examine the presence of gene expression signature of ferroptosis in psoriasis, we performed RNA sequencing (RNA-Seq) of psoriatic versus patient-matched perilesional skin and compared the differential transcriptional profile with the ferroptosis database, FerrDb (17). Our analysis revealed the upregulation of 3 ferroptosis markers (*Chac1*, *Slc40a1*, *Fth1*) and 29 ferroptosis drivers (including *Alox12B* and *Alox15B*, which encodes 15-LOX-2) as well as downregulation of 7 ferroptosis suppressors in psoriasis (Supplemental Table 2 and Figure 1B). Ingenuity Pathway Analysis revealed the enrichment of ferroptosis signaling pathway (*P* = 0.0013, *z* score = 2.04) in lesional versus perilesional skin of psoriasis patients (Figure 1C and Supplemental Table 3). Taken together, these results reveal the accumulation of ferroptosis-specific oxPE species in epithelial cells of inflammatory diseases involving the skin, lungs, and kidneys, and suggest a transcriptional profile of ferroptosis in the psoriatic epidermis.

### Pro-ferroptotic signals in psoriasis are enriched in K14^+^ KCs.

We reasoned that KCs undergoing ferroptosis may release proteins, lipids, and other signaling molecules that affect the neighboring KCs and modulate immune responses, thus eliciting inflammation. In support of this hypothesis, we observed that the replication rate of KCs increased upon exposure to factors released by other ferroptotic KCs, and that T lymphocytes and neutrophils were chemoattracted more to the ferroptotic versus control KCs (Supplemental Figure 2, B and C). Ferroptosis of the KCs could, therefore, promote psoriasis-associated epidermal proliferation and inflammation. To test for a similar effect in vivo, we checked whether topical application of pro-ferroptosis inhibitors of Gpx4 or the cystine-glutamate antiporter system x_c_^–^ would produce a psoriasis-like phenotype in mice. Applying RSL3 or erastin to ear and back skin of mice in vivo or ex vivo indeed increased ferroptosis-specific oxPE species in the epidermis, but not in the presence of Fer-1 (Supplemental Figure 2D and Supplemental Table 4). However, neither RSL3 nor erastin elicited histological epidermal changes or immune cell infiltration in the skin characteristic of psoriasis, while skin TNF-α and IL-1β expressions were elevated by RSL3 treatment (Supplemental Figure 2, E and F, and Supplemental Figure 3, A–D). We reasoned that this may be because of indiscriminate pro-ferroptotic activities of topical RSL3 and erastin on the KCs as well as immune and other types of skin cells, and while these agents increase pro-ferroptotic oxPE in the skin, they cannot fully recapitulate other elements of psoriasis such as KC proliferation and IL-23/Th17 inflammation. We therefore hypothesized that a specific subpopulation of the KCs may be executing ferroptosis in psoriasis.

Next, we proceeded to identify a subset of the KCs where the ferroptotic process may be occurring in psoriasis. We found that expression of *Gpx4*, a critical regulator of ferroptosis, was downregulated in psoriatic versus patient-matched healthy epidermis, consistent with published reports (Figure 1D) (11, 12, 18, 19). *Gpx4* was previously shown to be preferentially expressed in a subcluster of stem-like epidermal keratin 5/14–positive (K5/14^+^) KCs and in the progenitor K15^+^ KCs of follicular bulge/germinal layers — cycling KCs with the highest phospholipid content (14, 20–22). Indeed, we confirmed that in healthy epidermis K14^hi^ KCs in the basal/epibasal layers produced more Gpx4 versus K14^lo^ KCs of the more superficial spinous layer, with keratin 19^+^ KCs at the tips of the rete ridges containing the highest amounts of Gpx4 (Figure 1, E and F, and Supplemental Figure 4, A and B). By immunofluorescence analysis, Gpx4 levels were significantly decreased in psoriatic versus perilesional epidermis, consistent with previous reports (Figure 1, E and F) (12). Using additional complementary analytical methods, we confirmed that in psoriatic epidermis Gpx4 expression and protein levels were significantly reduced in EGFR^+^CD104^hi^CD49f^hi^ basal KC subpopulations relative to control nonlesional skin (Figure 1, G–I, and Supplemental Figure 4B).

In addition to the dysregulated glutathione-Gpx4 redox system, execution of ferroptosis depends on the peroxidation of polyunsaturated fatty acid–phospholipids (PUFA-PLs). These phospholipids are most abundant in the basal and lower spinous epibasal layers of the epidermis, rendering them potentially most vulnerable to ferroptosis (22). We previously showed that the generation of oxPE species by 15-LOX-2, which has high peroxidation activity to sn2-PUFA-PE and di-PUFA-PE species, enforces ferroptosis in the KCs (3, 23). Other prior studies of human skin demonstrated that basal and epibasal KCs are also the main producers of *Alox15B* (which encodes 15-LOX-2) in healthy epidermis (20, 24). In addition, *Alox15B* was shown to be upregulated by the basal KCs in psoriasis (21). Indeed, using confocal microscopy, we determined that 15-LOX-2 levels were increased in psoriatic epidermis versus perilesional skin, with a larger upregulation observed in the K5^hi^ basal/epibasal KCs versus K5^lo^ superficial spinous layer KCs (Figure 1, J and K, and Supplemental Figure 4C). Therefore, an imbalance between the enzymatic generation (via 15-LOX-2) and reduction (via Gpx4) of PUFA-PL hydroperoxides in psoriatic epidermis results in a measurable accumulation of oxidized lipids, which is most pronounced within the K5/14^hi^ KCs of the basal and deep spinous layers (Figure 1, L and M), and which suggests a probable KC pathway to ferroptosis.

**Figure 2:**
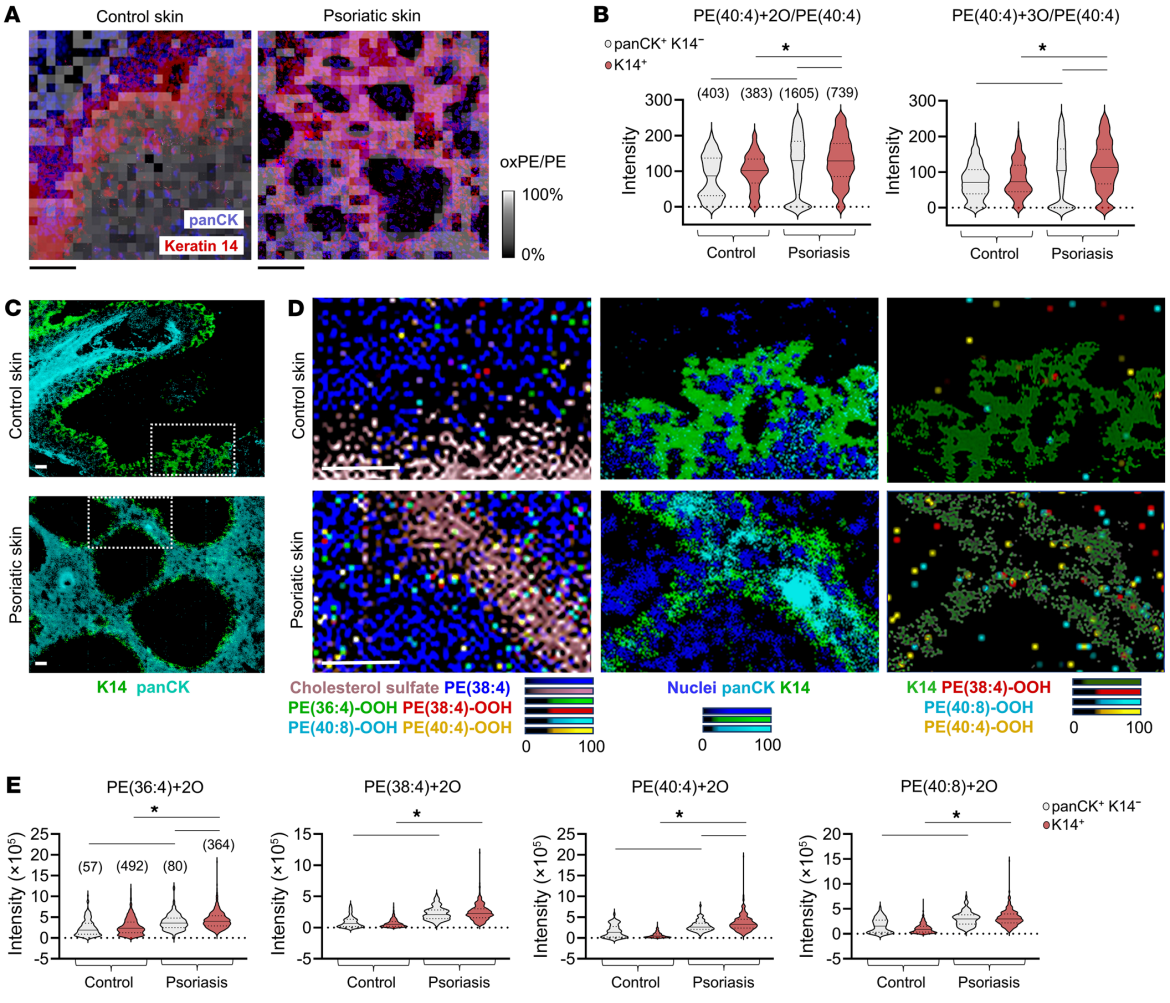
Elevated oxPE ferroptosis death signals in psoriatic epidermis are overrepresented within the K14^+^ KCs. (**A**) Overlay of MALDI-MS and IF imaging of human nonlesional (control) skin (left) and psoriatic skin (right). MALDI-MS ratiometric grayscale images (20 μm raster, negative ion mode) show the abundance ratio of detected *m*/*z* 826.6 to 794.6 ions, corresponding to PE(40:4)+2O and PE(40:4), respectively. Scale bars: 100 μm. PanCK, pan-cytokeratin. (**B**) MALDI-MS signal intensities (single pixel) of oxPE/PE [PE(40:4)+2O/PE(40:4), PE(40:4)+3O/PE(40:4)] in panCK^+^K14^–^ and K14^+^ regions of control and psoriatic epidermides. Numbers of pixels in each group are indicated over the first violin plot. (**C**) C_60_-SIMS imaging of K14^+^ and panCK^+^ cells in control skin (top) and psoriatic skin (bottom). Scale bars: 50 μm. Regions within dashed rectangles are shown magnified in **D**. (**D**) Overlay of panCK^+^ and K14^+^ epidermal regions (obtained by C_60_-SIMS) and lipids [cholesterol sulfate, PE, and oxPE species; obtained by (H_2_O)*_n_*-GCIB-SIMS] in control skin (top) and psoriatic skin (bottom). Color intensities representing each lipid species were adjusted to match a relative scale of 0 to 100 (color bars). Scale bars: 50 μm. (**E**) Single-cell (H_2_O)*_n_*-GCIB-SIMS signal intensities of select pro-ferroptotic oxPE species within panCK^+^K14^–^ and K14^+^ epidermal KCs in control versus psoriatic skin. Numbers of cells in each group are indicated over the first violin plot. Data are means ± SD. Two-tailed Student’s *t* test; *P < 0.05.

Because LC-MS–based redox lipidomics does not provide information on spatial distributions of analytes and thus cannot identify single ferroptotic cells, we used contemporary mass spectrometry imaging techniques of lipidomics analysis to map the distribution of oxPE in psoriatic epidermis. Initially, we used a matrix-assisted laser desorption/ionization mass spectrometry (MALDI-MS) imaging protocol, which permits the detection of peroxidation PUFA-PL substrates and products (3, 25, 26). To localize phospholipid signals within the epidermis, we stained skin sections with anti–pan-keratin and anti-K14 antibodies after they were imaged by MALDI-MS. When psoriatic and nonlesional epidermal KCs were compared by MALDI-MS/immunofluorescence microscopy, increased abundances of pro-ferroptotic oxPE species relative to their non-oxidized precursors were found in psoriasis within K14^+^ KCs (Figure 2, A and B). Our MALDI-MS imaging method is limited to a spatial resolution of about 20 μm, which is insufficient to distinguish individual cells. Therefore, we used our recently developed multiomics dual secondary ion mass spectrometry imaging at high spatial resolution (1–3 μm), employing a water gas cluster ion beam [(H_2_O)_*n*(*n*>24,000)_-GCIB] to localize lipids and their peroxidation products, and subsequent C_60_ beam to spatially profile proteins of interest on the same tissue section (Figure 2, C and D, and Supplemental Figure 5) (27, 28). The results of these single-cell studies confirmed enrichment in psoriasis of a subpopulation of K14^+^ KCs with significantly elevated levels of pro-ferroptotic oxPE products (Figure 2, D and E).

### Deletion of Gpx4 in a subset of K14^+^ KCs initiates and maintains psoriasiform disease in mice.

**Figure 3:**
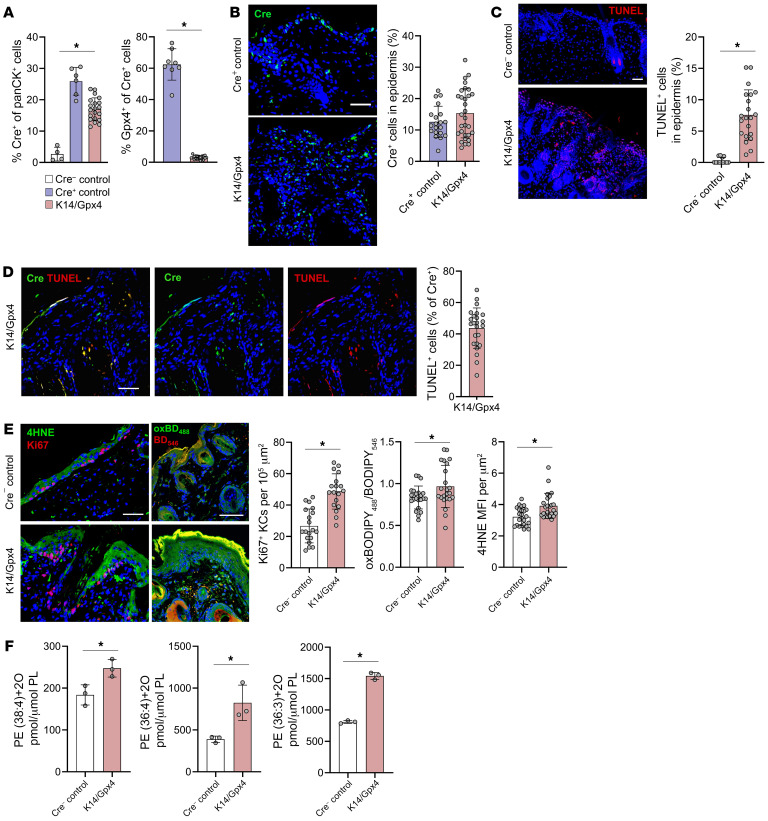
Gpx4 depletion in K14^+^ epidermal KCs of K14/Gpx4 mice triggers their ferroptosis. (**A**) Flow cytometry of the ear epidermis of K14/Gpx4, Cre^+^ control, and Cre^–^ control mice 25 days after TMX initiation. Each dot represents an individual mouse. (**B**–**E**) IF images of the skin of K14/Gpx4, Cre^+^ control, and Cre^–^ control mice 18 days after TMX initiation, and quantification of Cre^+^ (**B**) and TUNEL^+^ (**C**) KCs in epidermis, percentage TUNEL^+^ of Cre^+^ KCs (**D**), and Ki67^+^ KCs, epidermal oxC11-BODIPY_488_/BODIPY_546_ (MFI/MFI), and epidermal 4-HNE MFI per μm^2^ (**E**). Blue, DAPI; scale bars: 50 μm. *n* = 3 mice per group. (**F**) Levels of pro-ferroptotic death signals PE(38:4)+2O, PE(36:4)+2O, and PE(36:3)+2O in ear epidermis of K14/Gpx4 and Cre^–^ control mice 5 days after TMX initiation; *n* = 3 mice per group. Data are means ± SD. Two-tailed Student’s *t* test; *P < 0.05.

To better delineate the role of K14^+^ KC ferroptosis in psoriasis, we generated a tamoxifen-inducible (TMX-inducible) genetic mouse model, K14-CreERT^+/+^ Gpx4^fl/fl^ (referred to henceforth as K14/Gpx4), with Gpx4 depletion in K14-expressing KCs (Figure 3A and Supplemental Figure 6, A–F). In K14/Gpx4 mice, Cre-producing K14^+^ KCs constituted about 15% of the total epidermal cells (Figure 3, A and B). We used terminal deoxynucleotidyl transferase–mediated dUTP nick end labeling (TUNEL) to identify ferroptotic cells, as ferroptosis induced by Gpx4 depletion was previously shown to exhibit TUNEL positivity (29, 30). We found that approximately 7.6% of epidermal KCs of K14/Gpx4 mice were TUNEL^+^ versus approximately 0.31% TUNEL^+^ KCs in control mice (Figure 3C). All ferroptotic TUNEL^+^ KCs were Cre^+^, while approximately 44% of Cre^+^ cells were TUNEL^+^ (Figure 3D). The lipid peroxidation indicators oxBODIPY and 4-hydroxynonenal (4-HNE) as well as cell proliferation index (Ki67^+^ KCs) were significantly increased in the epidermis of K14/Gpx4 mice (Figure 3E). Consistent with psoriatic skin, LC-MS redox lipidomics revealed increased levels of several oxPE species in K14/Gpx4 versus control epidermis (Figure 3F and Supplemental Table 5). Orthogonal partial least squares discriminant analysis determined that among these, ferroptosis-specific oxPE death signals PE(36:4)+2O (Variable Importance in Projection [VIP] score = 1.292) and PE(38:4)+2O (VIP score = 1.288) contributed to the lipidome differences between K14/Gpx4 and control mice (Supplemental Figure 7, A and B, and Supplemental Table 5). Therefore, the K14/Gpx4 model actuates ferroptosis in a small subset of epidermal K14^+^ KCs.

**Figure 4:**
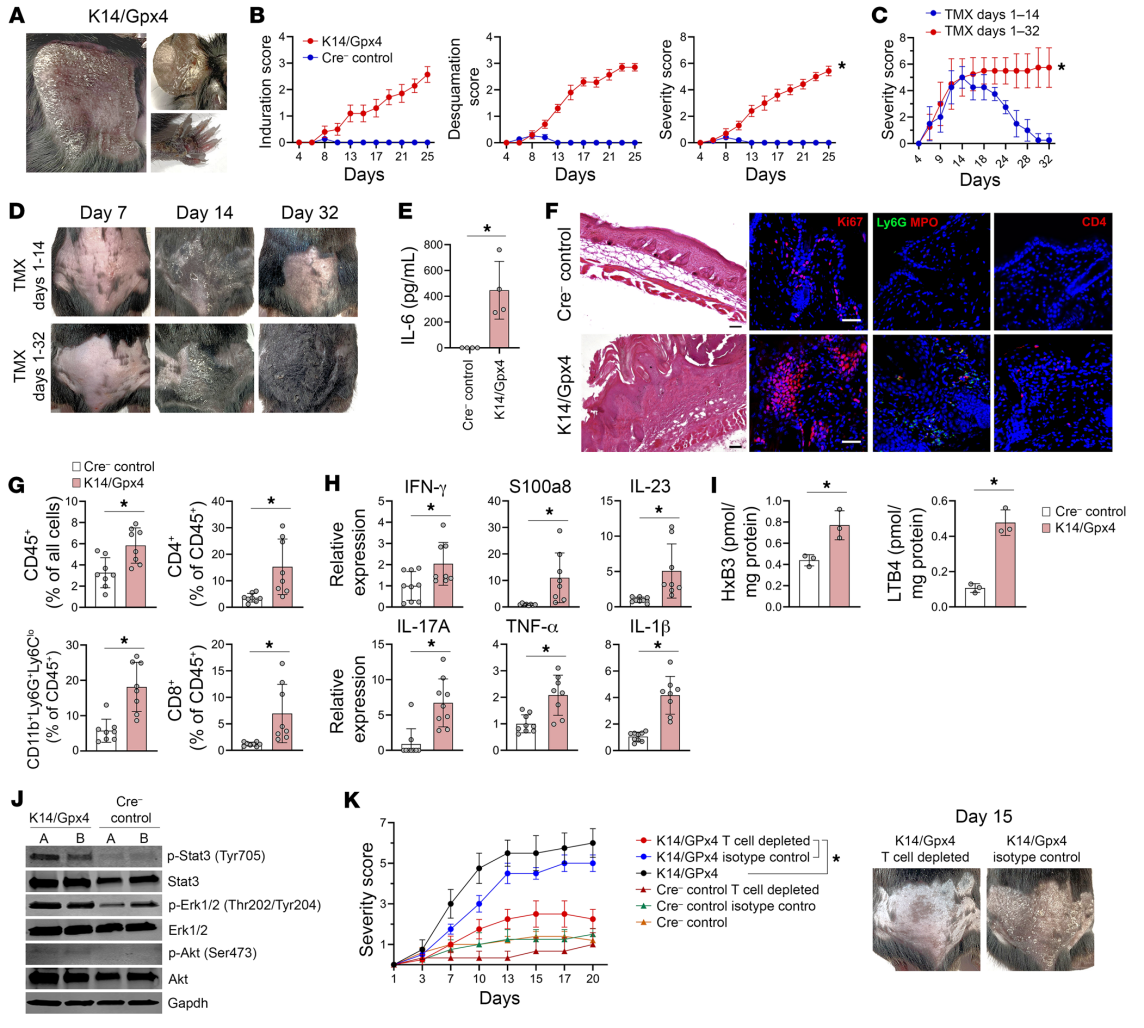
Gpx4 depletion in select K14^+^ KCs produces T cell–dependent psoriasiform inflammation. (**A**) Psoriasis-like skin changes in a K14/Gpx4 mouse 25 days after TMX initiation. (**B**) Skin induration, desquamation scores, and their sum (severity score) of K14/Gpx4 and Cre^–^ control mice as a function of days after TMX initiation; *n* = 10–15 mice per group. (**C** and **D**) Severity scores (**C**) and photographs (**D**) of K14/Gpx4 mice when TMX injections were continued over 32 days or stopped on day 14 after TMX initiation; *n* = 4 mice per group. (**E**) Serum IL-6 levels of K14/Gpx4 and Cre^–^ control mice (ELISA) 23 days after TMX initiation. (**F**–**I**) H&E and IF images (**F**), flow cytometry (**G**), quantitative real-time PCR (**H**), and LC-MS lipidomic analyses (**I**) of the skin of K14/Gpx4 and Cre^–^ control mice 23 days after TMX initiation. Blue, DAPI; scale bars: 50 μm. Each dot in **E**–**I** represents an individual mouse. (**J**) Western blot of Stat3, Erk1/2, and Akt phosphorylation in the skin of K14/Gpx4 and Cre^–^ control mice 15 days after TMX initiation; *n* = 2 mice per group. (**K**) Effects of antibody-mediated T cell depletion on the severity scores of K14/Gpx4 and Cre^–^ control mice as a function of time (days) after TMX initiation; *n* = 4 mice per group. Data are means ± SD. Two-way ANOVA (**B**, **C**, and **K**), 2-tailed Student’s *t* test (**E** and **G**–**I**); *P < 0.05.

We found that K14/Gpx4 mice developed a phenotype highly resembling psoriasis. Psoriasis-like changes were evident first on the skin at approximately day 10–12 after the initiation of TMX, and progressed to involve the majority of the skin surface, including ears, tail, and paws, while TMX was continued (Figure 4, A and B, and Supplemental Figure 7, C–F). TMX was required to maintain Gpx4 depletion. When TMX was stopped on day 14, Gpx4 in K14/Gpx4 epidermis returned to the level of control mice, and the psoriatic phenotype gradually disappeared (Figure 4, C and D, and Supplemental Figure 6, G and H). This suggests that ferroptosis of a fraction of K14^+^ KCs is required to maintain a new epidermal steady state with simultaneous increases in KC death and proliferation rates. K14/Gpx4 mice also developed signs of psoriasis-associated systemic inflammation — elevated serum proinflammatory cytokines (e.g., IL-6; Figure 4E), an increase of splenic CD4^+^ and CD8^+^ T cells, CD4^+^ T cell infiltration of the hind-paw synovium, neutrophilic infiltration of the liver and the lungs (Supplemental Figure 8, A–D). Analyses of the skin of K14/Gpx4 mice revealed parakeratosis and acanthosis with elevated epidermal proliferation index, mixed dermal immune cell infiltrate including T lymphocytes, clusters of neutrophils in the epidermis, and upregulation of psoriasis-associated cytokines and lipid mediators (Figure 4, F–I, and Supplemental Figure 8, E and F). Activation of Stat3, Erk, and Akt, signaling pathways implicated in psoriasis and indicative of KC proliferation, was detected in K14/Gpx4 epidermis (Figure 4J). Importantly, antibody-mediated depletion of T cells prevented TMX-induced psoriatic phenotype in K14/Gpx4 mice (Figure 4K and Supplemental Figure 8, G–J). Taken together, our results suggest that ferroptosis of a subset of K14^+^ KCs, induced by Gpx4 deficiency, is sufficient to initiate and maintain a T cell–dependent psoriasis-like phenotype in the K14/Gpx4 model.

### Transcriptional profile concordance between K14/Gpx4 model and human psoriasis.

**Figure 5:**
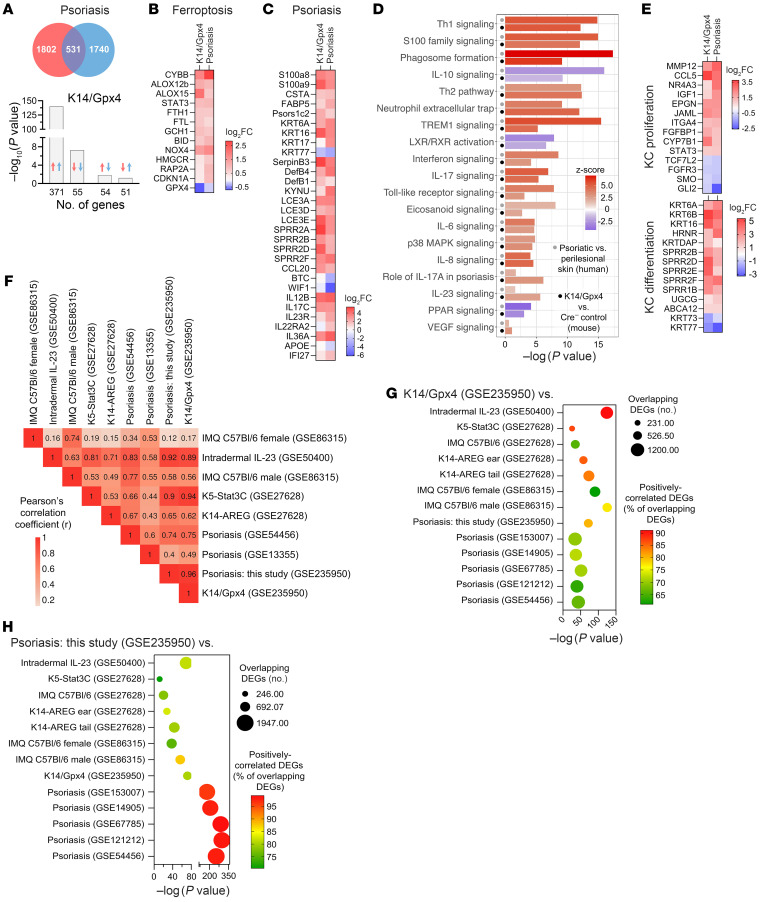
K14/Gpx4 model recapitulates transcriptional profile of psoriasis. (**A**) Comparison of DEGs (*P* < 0.05, |FC| > 1.5) in psoriatic skin (vs. patient-matched perilesional skin; *n* = 3 patients) with DEGs in K14/Gpx4 skin (vs. Cre^–^ controls; *n* = 3 mice per group) using Illumina Correlation Engine. (**B** and **C**) Changes in the expression of ferroptosis-associated (**B**) and psoriasis-associated (**C**) genes (*P* < 0.05, |FC| > 1.5) in psoriatic versus patient-matched perilesional skin and K14/Gpx4 versus Cre^–^ control mouse skin (18 days after TMX initiation). (**D** and **E**) Ingenuity Pathway Analysis (IPA) (**D**) and Gene Ontology (**E**) enrichment analyses using DEGs of psoriasis versus patient-matched perilesional skin and DEGs of K14/Gpx4 versus Cre^–^ control mouse skin. (**F**) Pearson’s correlation coefficients from pairwise comparisons of transcriptional profiles of 3 human psoriasis datasets (including this study) and 5 mouse models of psoriasis (including K14/Gpx4) based on *z* scores of 19 psoriasis-associated pathways shown in **D**. (**G** and **H**) Correlation analyses of the overlapping DEGs between K14/Gpx4 model (vs. Cre^–^ control) (**G**) or human psoriasis (lesional vs. patient-matched perilesional skin) (**H**) and select published studies on human psoriasis and murine models; Fisher’s exact test, Illumina Correlation Engine.

We next asked whether the K14/Gpx4 model recapitulates the gene expression profile of psoriasis and performed RNA-Seq of K14/Gpx4 lesional skin versus Cre^–^ control skin (Supplemental Table 6). Comparison of the K14/Gpx4 transcriptional profile with that of human psoriatic skin revealed 531 shared differentially expressed genes (DEGs), 426 of which showed positive correlation (Figure 5A), including those genes associated with psoriasis and ferroptosis (Figure 5, B and C, and Supplemental Table 6) (31). Multiple enriched psoriasis-associated signaling pathways were also detected in the Krt14/Gpx4 model (Figure 5D and Supplemental Table 7). In addition, Gpx4-depleted mouse skin transcriptome exhibited an enrichment of signaling pathways related to KC proliferation (GO:0050679, fold change = 1.7, *P* = 2.91 × 10^–4^, FDR = 6.14 × 10^–3^) and KC differentiation (GO:0030216, fold change = 2.9, *P* = 1.15 × 10^–10^, FDR = 8.65 × 10^–9^), consistent with human disease (Figure 5E and Supplemental Table 8).

We also compared how the K14/Gpx4 model recapitulates the gene expression pattern of psoriasis relative to published studies of human disease (18, 32), and relative to other mouse models of psoriasis: topical imiquimod (IMQ) (33, 34), K14-AREG (35, 36), K5-Stat3C (36, 37), and intradermal IL-23 (idIL-23) (38, 39), which vary in their transcriptional similarities to psoriasis (Supplemental Figure 9, A and B, and Supplemental Table 9) (36). Based on the *z* scores of 19 selected canonical psoriasis-associated pathways, K14/Gpx4 showed the strongest correlation to human psoriasis datasets (*r* = 0.75–0.96), as well as to K5-Stat3C (*r* = 0.94) and idIL-23 (*r* = 0.89) models, and weaker correlation to K14-AREG (*r* = 0.62) and IMQ (*r* = 0.17–0.56) models (Figure 5F, Supplemental Figure 9, A and B, and Supplemental Table 9). Pairwise correlation analyses of overlapping DEGs between K14/Gpx4 and published datasets (18, 34, 36, 39–43) were also performed using Fisher’s exact test, revealing that the K14/Gpx4 transcriptional profile was closest to the idIL-23 model (Figure 5G and Supplemental Table 10). Among the considered mouse models, K14/Gpx4 and idIL-23 showed the most significant correlations with the human psoriasis transcriptome (Figure 5H and Supplemental Table 10).

Next, we compared transcriptional profiles of K14/Gpx4 lesional epidermal KCs and published single-cell RNA-Seq of KCs isolated from human psoriatic epidermis (Supplemental Table 11) (21, 44). Transcriptional analysis of K14/Gpx4 epidermis revealed multiple shared regulatory pathways with psoriatic KCs related to inflammation, cell proliferation and metabolism, redox dysregulation, and programmed cell death pathways (Supplemental Figure 10, A–C, and Supplemental Table 11). Gene expression of K14/Gpx4 versus Cre^–^ control epidermis demonstrated significant correlation to the psoriatic KC population as a whole, as well as to distinct epidermal KC subpopulations of psoriatic skin determined previously by single-cell transcriptomics (Supplemental Figure 10, A–C, and Supplemental Table 11) (21, 44). Overall, these results demonstrate concordance of transcriptional changes in K14/Gpx4 skin and human psoriasis.

### Psoriasiform dermatitis of K14/Gpx4 model is effectively treated with cytokine-directed immunoglobulins and ferroptosis inhibitors.

**Figure 6:**
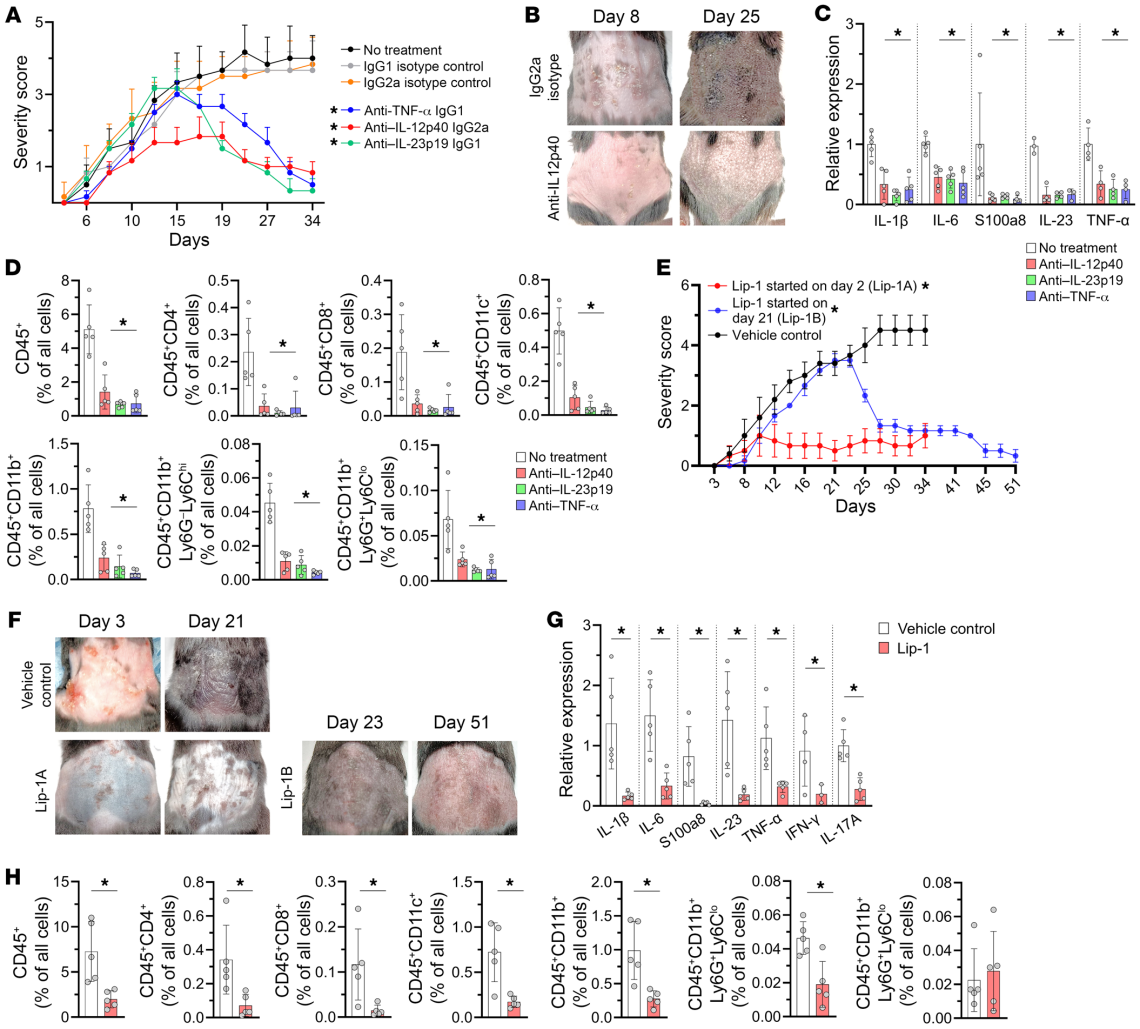
Psoriasiform inflammatory phenotype of K14/Gpx4 model responds to anti–IL-12/IL-23/TNF-α and anti-ferroptosis therapies. (**A**) Severity scores of K14/Gpx4 mice treated with anti–IL-12p40, anti–IL-23p19, or anti–TNF-α antibodies or IgG1/IgG2a isotype control antibodies, in conjunction with TMX; *n* = 6 mice per group, *P < 0.05 vs. vehicle control and respective isotype control. (**B**) Photographs of K14/Gpx4 mice treated with anti–IL-12p40 antibodies or isotype control antibodies on days 8 and 25 after TMX initiation. (**C** and **D**) Relative expression of IL-1β, IL-6, S100a8, IL-23, and TNF-α (RT-qPCR) (**C**) and percentages of immune cells (flow cytometry) (**D**) in skin of K14/Gpx4 mice treated with anti–IL-12p40, anti–IL-23p19, or anti–TNF-α antibodies or vehicle control 35 days after TMX initiation; *n* = 5 mice per group, *P < 0.05 vs. no-treatment control. (**E**) Severity scores of K14/Gpx4 mice treated with Lip-1 or vehicle control, in conjunction with TMX. Lip-1 was started on either day 2 (Lip-1A) or day 21 (Lip-1B); *n* = 5–6 mice per group, *P < 0.05 vs. control (days 3–34 for Lip-1A, days 25–34 for Lip-1B). (**F**) Photographs of K14/Gpx4 mice treated with Lip-1 or vehicle control. Shown are days 3 and 21 for Lip-1A and days 23 and 51 for Lip-1B after TMX initiation. (**G** and **H**) Relative expression of IL-1β, IL-6, S100a8, IL-23, TNF-α, IFN-γ, and IL-17A (RT-qPCR) (**G**) and percentages of immune cells (flow cytometry) (**H**) in skin of K14/Gpx4 mice treated with Lip-1A or vehicle control 35 days after TMX initiation; *n* = 5 mice per group, *P < 0.05 vs. control. Data are means ± SD. Two-way ANOVA (**A** and **E**), 1-way ANOVA (**C** and **D**), 2-tailed Student’s *t* test (**G** and **H**). Each dot in **C**, **D**, **G**, and **H** represents an individual mouse.

Psoriasis is a T cell–dependent disease that is usually successfully treated with therapies involving the inhibition of IL-12/IL-23/IL-17 and TNF-α axes. Therefore, we next assessed the efficacies of these immunoglobulin-based antipsoriasis treatment approaches in K14/Gpx4 mice (Supplemental Figure 11, A and B). IL-12p40–, IL-23p19–, and TNF-α–targeting antibodies, administered in conjunction with TMX, effectively reversed early psoriasiform skin changes in K14/Gpx4 mice and maintained low severity scores for the duration of treatment (Figure 6, A and B). One month after the initiation of immunoglobulin-based therapies, markers of psoriatic inflammation were significantly reduced in the skin of K14/Gpx4 mice versus controls (Figure 6, C and D). Mice receiving therapeutic antibodies demonstrated no signs of systemic inflammation or distress 2 months after the initiation of TMX, and appeared healthy compared with mice receiving isotype control antibodies. In addition, administrations of a ferroptosis inhibitor, Lip-1, not only prevented the emergence of psoriasis, but also successfully treated an established psoriasiform dermatitis and systemic inflammation of K14/Gpx4 mice receiving TMX (Figure 6, E–H). Oxidized lipid content in the epidermis of K14/Gpx4 mice, measured by 4-HNE immunostaining, was significantly reduced by Lip-1 and the therapeutic antibodies, although to a lesser degree by the latter (Supplemental Figure 11C). Altogether, these data indicate that the ferroptotic process within a subset of K14^+^ KCs engages inflammatory mechanisms implicated in psoriasis pathogenesis. Furthermore, our K14/Gpx4 model demonstrates that both ferroptosis of some KCs and subsequent T cell–mediated immune responses are required to maintain epidermal lipid redox dysregulation and chronic inflammation of psoriasis.

## Discussion

Here, we uncover a previously unappreciated mechanism of the initiation and maintenance of chronic multiorgan inflammation based on the occurrence of ferroptosis in select epithelial cells. More specifically, we show that the K14/Gpx4 model, which enforces psoriasis-associated sporadic pro-ferroptotic phospholipid peroxidation in a relatively small fraction of KCs, recapitulates most of the phenotypic, immunological, and multiomic hallmarks of psoriasis, including an effective response to current standard biological therapies. Our results provide direct evidence that redox dysregulation that leads to immunogenic ferroptotic epithelial cell death can be a pathogenic trigger and a driver of chronic systemic inflammatory diseases.

Ferroptosis of the KCs results in a plethora of oxidized free fatty acids and oxPL species with signaling potential to neighboring KCs and immune cells. We have shown that the ferroptotic process in a subset of KCs increases the proliferation rate of other KCs and causes dysregulation of epidermal differentiation. This may explain the seemingly paradoxical co-occurrence of programmed cell death and hyperproliferation in the epithelium. The signaling mechanisms behind this process, which are yet to be determined, may involve phospholipids, lysophospholipids (lysoPLs), and their metabolic derivatives generated and released during ferroptosis, including those previously shown to promote epidermal proliferation and acanthosis of psoriasis (e.g., lysophosphatidic acid) (10, 45, 46). In addition, ferroptosis may not strictly be a cell-autonomous process, and may spread to adjacent cells (47). Thus, vulnerabilities of some KCs to ferroptosis, even if a result of a transient stimulus, may potentially initiate a pro-ferroptotic wave that propagates along an epithelial layer, maintaining a chronic inflammatory process. While in psoriatic epidermis the highest levels of oxPE species, ferroptotic death signals, and their substrates were observed in K14^+^ KCs and correlated with decreased Gpx4 and increased 15-LOX, we cannot rule out that ferroptosis may also occur within K14^lo^ KCs residing in a more superficial stratum granulosum.

We show here that ferroptosis of a fraction of KCs is sufficient to activate Th1 and IL-23/Th17 responses and produce psoriasiform dermatitis. However, we also found that both KC ferroptosis and T cell responses triggered by ferroptosis are required for the maintenance of psoriasiform dermatitis in the K14/Gpx4 model. Such an epithelial-immune microenvironment dysregulation is at the center of chronic inflammatory diseases of the skin and other organs (5, 48), and our K14/Gpx4 model successfully integrates KC-centric and immune hypotheses of psoriasis pathogenesis. As we demonstrate, it is possible that factors released by the ferroptotic KCs attract and activate neutrophils, T lymphocytes, and other immune cells. Oxidized lipids and their protein adducts produced in the ferroptotic KCs may be taken up by the adjacent KCs or dendritic or other antigen-presenting cells and serve as neolipid antigens in the activation of the T cells (49). The immune response to a sporadic KC ferroptosis may subsequently impose a feedforward signaling inflammatory loop involving other KCs, resulting in immune-mediated ferroptosis of new KCs and epidermal proliferation. Ferroptosis has been shown to produce immune activation as well as immunosuppression, largely depending on the type of cell undergoing the programmed death, the mechanism of cell death induction, the tissue microenvironment, and other factors (50–52). It remains to be determined precisely how ferroptosis of KCs initiates and maintains skin and systemic inflammatory responses, and whether these mechanisms are organ specific or conserved in other inflammatory diseases such as asthma, lupus erythematosus, and ulcerative colitis.

We have shown that within human epidermis Gpx4 is enriched in basal/epibasal K14^+^ KCs, and that Gpx4 is downregulated in psoriatic epidermis, rendering those KCs vulnerable to ferroptosis. Constitutive knockout of Gpx4 in K14^+^ KCs hinders postnatal hair follicle development and produces cutaneous inflammation (53). Factors that lead to diminished levels of Gpx4 in psoriasis remain unknown. Vitamin D, essential in skin homeostasis, is routinely used to treat psoriasis, and was previously shown to inhibit ferroptosis and increase Gpx4 expression (54, 55). It is possible that documented insufficiency of vitamin D in psoriasis patients may lead to a pro-ferroptotic redox imbalance in the KCs (56). Selenium has also been implicated in the regulation of Gpx4 expression and protection from ferroptosis in different organs (57, 58). While the link between selenium levels in the skin and psoriasis severity is controversial, selenium has been reported to improve skin inflammation (59). In addition, low selenium level in serum positively correlates with the severity of airway and gastrointestinal inflammation, and its supplementation has also shown benefits in treatments of asthma and inflammatory bowel disease (60, 61). Ferroptosis vulnerability in epithelial cells arises from varying levels or activities of other enzymes, such as lipoxygenases that generate oxPE death signals. Here, we show that 15-LOX-2 levels are increased in K5^+^ KCs of psoriatic epidermis (which coexpress K14). Consistent with these findings, we previously showed that increased 15-LOX-1 in bronchial epithelia and nasal polyp basal epithelial cells of patients with asthma contributes to redox dysregulation, inflammation, and worse clinical outcomes (1, 62). Therefore, organ-specific investigations are required to elicit factors that initiate epithelial ferroptosis and drive chronic inflammatory processes in various diseases.

Restoration of physiological tissue homeostasis is the principal aim of therapeutic and curative interventions, and, despite major progress in the use of biological immunomodulators, complete understanding of the pathogenesis of chronic inflammatory diseases affecting the skin and other organs remains a considerable medical challenge. Elucidation of the pathological processes within disease-initiating cells of the epithelial-immune microenvironment will lead to new strategies for prevention and treatment. In that context, our findings that psoriasis may be driven by the dysregulation of oxidized phospholipids in a subset of K5/14^+^ KCs shift the paradigm of psoriasis as primarily an immune-mediated disease and open new opportunities for preventative and therapeutic approaches that target epithelial ferroptosis in multiple chronic inflammatory conditions.

## Methods

### Sex as a biological variable.

Analyses of human psoriasis tissues were performed on skin samples from equal numbers of men and women, with ages ranging from 27 to 43. Both male and female mice were used in every experimental group of the study.

### Animal models.

Transgenic KRT14-cre/ERT^20Efu/J^ mice (stock 005107), Gpx4tm1.1^Qra/J^ mice (stock 027964), and C57BL/6 female mice were purchased from The Jackson Laboratory. KRT14-Cre and GPX4-floxed mice were crossed to generate KRT14-CreERT^+/+^ GPX4^fl/fl^ (K14/Gpx4), KRT14-CreERT^–/–^ GPX4^fl/fl^, and KRT14-CreERT^+/+^ GPX4^wt/wt^ mouse lines. Genotyping was performed on DNA extracted from tail snips using a direct lysis buffer kit (Viagen Biotech 102-T). Primers for the detection of *Krt14* and *Gpx4* transgenes are listed in Supplemental Table 12. Homozygous and hemizygous KRT14-Cre were differentiated using primers and protocols specified by The Jackson Laboratory (Supplemental Table 13). For topical treatment with RSL3 or erastin, 6- to 8-week-old female C57BL/6 mice were treated on dorsal shaved skin and ear with 120 μL of RSL3 (10 μM; Sigma-Aldrich), Lip-1 (2 μM; Cayman Chemical), or erastin (10 μM; Sigma-Aldrich) in 70% ethanol twice per day for 2 weeks (Supplemental Table 14). All chemicals were dissolved in DMSO and 70% ethanol, and DMSO in 70% ethanol was used as a vehicle control. For ex vivo treatment, skin pieces harvested from back or ear were incubated in 12-well plates with vehicle control (DMSO/EtOH), RSL3 (10 μM; Sigma-Aldrich), erastin (10 μM; Sigma-Aldrich), RSL3 plus Fer-1 (10 μM; Sigma-Aldrich), or erastin plus Fer-1 (10 μM; Sigma-Aldrich) in dermal basal media for 15 hours. Epidermides were then separated and flash-frozen for subsequent LC-MS redox lipidomics analyses. All therapeutic antibodies — anti–IL-12p40 (IgG2a), anti–IL-23p19 (IgG1), and anti–TNF-α (IgG1) — and their respective isotype control antibodies were purchased from Bio X Cell (Supplemental Table 15), and administered i.p. according to the schematic shown in Supplemental Figure 11A. Lip-1 was administered i.p. (15 mg/kg; Cayman Chemical) and topically (8 mM in 70% ethanol; Selleck Chemicals) according to the schematic shown in Supplemental Figure 11B. To deplete T cells, anti-CD4 and anti-CD8a or isotype control antibodies (Bio X Cell; Supplemental Table 15) were injected i.p. according to the schematic shown in Supplemental Figure 8H. CD4^+^ and CD8^+^ T cell depletions were confirmed in the blood and the spleen. To induce Cre recombination, tamoxifen (TMX; Sigma-Aldrich T5648) dissolved in corn oil (20 mg/mL; Sigma-Aldrich C8267) was injected i.p. (75 mg/kg). Severity score (desquamation score + induration score) was assessed every other day according to the scale shown in Supplemental Figure 7, E and F. All Cre^–^ controls received TMX treatments identical to those of the Cre^+^ groups.

### Primary cells and cell lines.

Primary adult human epidermal keratinocytes (HEKa) (ATCC PCS-200-011) were cultured in dermal cell basal medium (ATCC PCS-200-030) or keratinocyte growth factor kits (ATCC PCS-200-040), containing antibiotic and antimycotic solution (Sigma-Aldrich A5955). HaCaT cells (AddexBio, catalog t0020001) were cultured in RPMI 1640 medium supplemented with l-glutamine (4 mmol/L; Gibco), penicillin (100 U/mL), streptomycin (100 mg/mL), amphotericin B (0.25 mg/mL; Sigma-Aldrich), HEPES (10 mmol/L; Lonza), 2-mercaptoethanol (55 mmol/L; Gibco), and 10% heat-inactivated FBS (Atlanta Biologicals). All cells were cultured at 37°C and maintained by passaging biweekly, and assays were performed at 70% confluence at or below passage 5. Trypsin-EDTA (ATCC PCS-999-003) and Trypsin Neutralizing Solution (ATCC PCS-999-004) were used to detach the primary cells.

### Human tissue.

Tissue samples from pulmonary airways of patients with cystic fibrosis and asthma and urine cell pellets from patients with renal failure were collected as described previously (1–3). When required, epidermal sheets were separated from the skin by first rinsing with PBS and removing fat. The skin was then cut to an appropriate surface area and incubated in dispase solution (1 U/mL) in DMEM/F-12 (Stem Cell Technologies 07923) for 7 hours at 37°C. Finally, intact epidermal sheets were separated from the dermis using forceps while being visualized with an optical microscope.

### Cell viability assay.

HEKa cells were incubated in 96-well plates (10^4^ cells per well) for 12 hours at 37°C and treated with erastin (2 μM; Sigma-Aldrich), RSL3 (10 μM; Sigma-Aldrich), Fer-1 (10 μM; Sigma-Aldrich), Lip-1 (1 μM; Cayman Chemical), baicalein (5 μM; Tocris), or vehicle control for 6 hours at 37°C. Cell viability was assessed with a CellTiter 96 Aqueous Cell Proliferation Assay kit (MTS, Promega G3582). Absorbance was measured at 490 nm with a SpectraMax iD5 Multi-Mode Microplate Reader (Molecular Devices).

### Proliferation assay.

HEKa cells at 60% confluence were treated with 10 μM of RSL3 or vehicle control (containing DMSO) for 6 hours at 37°C, then washed 3 times with PBS and incubated for 24 hours in a complete dermal cell basal medium. Supernatants were collected and passed through 0.45 μm filters, and added to fresh HEKa cells in 96-well plates (10^4^ cells per well). The BrdU Cell Proliferation kit (Millipore 2750) was used after 24 hours, and data acquired with a SpectraMax iD5 Multi-Mode Microplate Reader (Molecular Devices) at 450/550 nm.

### Leukocyte transmigration assay.

Epidermides of C57BL/6 mice were treated with RSL3 (5 μM; Sigma-Aldrich) in DMEM for 6 hours, washed 3 times with PBS, and incubated in DMEM at 37°C. Alternatively, epidermides of TMX-treated K14/Gpx4 mice and Cre^–^ controls were incubated in DMEM at 37°C. After 24 hours, all conditioned media were collected from the plates containing epidermides and stored for subsequent assays. Spleens and bone marrow of C57BL/6 mice were gently dissociated and passed through 70 μm cell strainers, and single-cell suspensions were spun down in chilled FACS buffer for 10 minutes at 400*g*. T cells were isolated from spleens using the Pan T-cell Isolation Kit II (Miltenyi Biotec 30-095-1), and neutrophils were isolated from bone marrow using the Neutrophil Isolation Kit (Miltenyi Biotec 130-097-658). Cells were counted using a DeNovix CellDrop BF cell counter, and applied on top of the Transwell 6.5 mm polycarbonate membranes, 5 μm pore size (Corning 3421), 3 × 10^4^ per well of T cells or 2 × 10^5^ per well of neutrophils in 100 μL. Bottom chambers were filled with 400 μL of epidermis-conditioned media. After 4 hours, cells in the bottom chambers were washed, stained and counted by flow cytometry (60 seconds per well).

### Flow cytometry.

To identify immune cell populations, skin was harvested, minced, and incubated in DMEM (Corning 10-013CV) containing collagenase D (1 mg/mL; Sigma-Aldrich C0130), hyaluronidase (10 mg/mL; Sigma-Aldrich H3884), and DNase (1 mg/mL; Sigma-Aldrich DN25) for 45 minutes at 37°C, and passed through a 70 μm cell strainer (Falcon 352350). For keratinocyte phenotyping, skin was incubated in DMEM containing 0.25% trypsin (Gibco 15090-046), collagenase D (1 mg/mL; Sigma-Aldrich), DNase (1 mg/mL; Sigma-Aldrich), and hyaluronidase (10 mg/mL; Sigma-Aldrich) for 30 minutes at room temperature and passed through a 70 μm cell strainer (Falcon 352350). Single-cell suspensions were washed with PBS and stained in FACS buffer (MACS buffer, Miltenyi Biotec 130-091-221) with the primary antibodies (Supplemental Table 16) and the Fixable Viability Dye (FVD) eFluor 780 (Invitrogen 65-0865-18) for 1 hour at 4°C. For the intracellular protein staining, cells were treated with Reagent A (Fix & Perm Cell Permeabilization Kit, Invitrogen GAS002) for 15 minutes at room temperature, then stained with the primary antibodies (Supplemental Table 16) for 45 minutes at 4°C in Reagent B (Invitrogen). For unconjugated primary antibodies, appropriate secondary antibodies were incubated in Reagent B (Invitrogen) for 45 minutes at 4°C. Flow cytometry was performed on a BD LSRFortessa X20 Cytometer (BD Biosciences), and data were analyzed using FlowJo software (BD Biosciences).

### Fluorescence-activated sorting of human keratinocytes.

Human epidermides were minced and digested for 45 minutes with 0.25% trypsin (Gibco 15090-046) and 5 mM EDTA (Thermo Fisher Scientific 15694) in DMEM at a mixing frequency of 1,200 rpm on an Eppendorf Thermo Mixer at 37°C. Digested tissue was passed through a 100 μm cell strainer to obtain a single-cell suspension, and washed in FACS buffer (MACS buffer, Miltenyi Biotec 130-091-221). Cells were stained with FVD (eBioscience 65-0865-14), anti-CD45, anti-EGFR, anti-CD104, and anti-CD49f antibodies (Supplemental Table 16) for 30 minutes at 4°C, then washed in FACS buffer. Cell sorting was performed with an Aurora CS Cell Sorter (Cytek Biosciences) using a 100 nm nozzle.

### Real-time PCR.

RNA was extracted from the whole skin or epidermis sample and stored in RNAlater (Invitrogen AM7024). Samples were homogenized in TRIzol reagent (Thermo Fisher Scientific 15596018) using a Bullet Blender Homogenizer (Next Advance) at speed 8 for 5 minutes in Navy Rhino tubes (Next Advance NAVYR1). RNA was isolated using TRIzol reagent per the manufacturer’s protocol and quantified using a DeNovix DS-11 spectrophotometer. Total RNA (2 μg) was converted to cDNA using a QuantiTect Reverse Transcription Kit (QIAGEN 205314). Quantitative real-time PCR (RT-qPCR) was performed using TaqMan probes labeled with FAM-ZEN/IBFQ (Integrated DNA Technologies; Supplemental Table 17), an endogenous control (β-actin) labeled with VIC-MGB-PL (Applied Biosystems.), and a TaqMan Fast Advance Master Mix (Applied Biosystems 44-445-57). For some gene targets, RT-qPCR was performed with Fast SYBRGreen Master Mix (Applied Biosystems 4385612) using appropriate primers (Supplemental Table 18). *GAPDH* was used as endogenous control. Reactions were run and analyzed on a StepOnePlus Real-Time PCR System (Applied Biosystems). Relative gene expression was calculated and normalized based on the 2^–ΔΔCt^ method.

### Immunofluorescence microscopy.

For C11-BODIPY (581/591) staining, fresh skin tissue was immersed in 2 μM C11-BODIPY solution (Thermo Fisher Scientific D3861) in PBS for 30 minutes at 37°C and washed twice with PBS before fixation. Skin samples were fixed in 2% paraformaldehyde (PFA) for 2 hours, then incubated in 30% sucrose in PBS for 24 hours. Tissues were frozen in 2-methylbutane (Sigma-Aldrich) in liquid nitrogen, embedded in Tissue-Plus OCT Compound (Fisher Scientific 23-730-625), and processed into 12-μm-thick sections. Tissue sections were permeabilized with 0.1% Triton X-100 (Sigma-Aldrich T9284) in PBS for 10 minutes, blocked with 2% BSA (Sigma-Aldrich A9647) and 5% goat serum (Gibco 16210-072) in PBS for 1 hour, and washed with 0.5% BSA in PBS containing 0.1% Triton X-100. Immunostaining was performed for 16 hours at 4°C with primary antibodies, followed by secondary antibodies (Supplemental Table 19) for 1 hour at room temperature, followed by nuclear DAPI stain (1 mg/mL; Sigma-Aldrich). For TUNEL staining a Click-iT Plus TUNEL Assay kit (Invitrogen C10618) was used. Briefly, tissue sections were fixed with 4% PFA for 15 minutes at 37°C, permeabilized with proteinase K, and incubated with TdT reaction buffer for 10 minutes at 37°C, followed by TdT reaction mixture for 1 hour at 37°C. After washing with 3% BSA in 0.1% Triton X-100 in PBS, tissue sections were incubated with a Click-iT Plus TUNEL reaction cocktail for 30 minutes at 37°C in the dark, followed by nuclear DAPI stain. Tissue sections were mounted in Gelvatol medium (Sigma-Aldrich) and imaged using a Nikon A1 confocal microscope. Image analyses were performed with NIS-Elements AR 4.40 software (Nikon).

### Western blotting.

Total protein was extracted from the whole skin or epidermis using T-PER Tissue Protein Extraction Reagent (Thermo Fisher Scientific 78510) containing protease inhibitors (Thermo Fisher Scientific 78425) and phosphatase inhibitors (Cell Signaling 5870S) with a handheld homogenizer on ice. Tissue extracts were incubated on ice for 30 minutes, sonicated, and centrifuged at 1,200*g* for 10 minutes. Protein concentrations were measured using a BCA kit (Thermo Fisher Scientific 23225). Samples were heated in a loading buffer at 80°C for 5 minutes, and 60 μg of protein per sample was run on a 15% SDS polyacrylamide gel, then transferred to a PVDF membrane. Membranes were blocked with Odyssey Blocking Buffer (LI-COR 927-70001) for 1 hour at room temperature, incubated with primary antibodies (Supplemental Table 20) for 16 hours at 4°C, washed 3 times with PBST buffer for 10 minutes, and incubated with conjugated secondary antibodies (LI-COR) for 1 hour at room temperature. Immunoblotted membranes were scanned on an Odyssey Scanner (LI-COR). Densitometry measurements on the immunoreactive bands were performed using ImageJ software (NIH), with β-actin and GAPDH as internal controls.

### ELISA.

Whole blood was collected from K14/Gpx4 or Cre^–^ control mice by cardiac puncture, and serum was separated by centrifugation at 1,000*g* for 10 minutes at 4°C in a microtainer blood collection tube (BD Biosciences 365967). Measurements of IL-6 in serum (diluted 1:10) were performed using a mouse IL-6 ELISA kit (BD Biosciences 555240) and a SpectraMax iD5 Multi-Mode Microplate Reader (Molecular Devices), and the concentrations were determined using SoftMax Pro 7.1 software (Molecular Devices).

### RNA sequencing.

Total RNA from mouse skin, human skin, or mouse epidermis was isolated using RNeasy Fibrous Tissue Mini Kit (QIAGEN 74704). Quality and concentration of RNA was determined with a Qubit fluorometer (Thermo Fisher Scientific) and an Agilent 2100 Bioanalyzer. Samples with 100 ng total RNA and RNA integrity numbers between 5 and 8.6 were used to prepare cDNA libraries. Sequencing libraries were constructed using Roche Kapa RNA HyperPrep with RiboErase (HMR) to run on Illumina platforms (KR1351, v4.21; 8098140702) with a read length of 2 × 76 bp. Approximately 703,537,213 total paired-end reads for mouse skin and approximately 186,661,371 total paired-end reads for mouse epidermis and human skin were obtained per sample (150 cycles; Illumina Next Seq 500/550 v2.5 Mid Output and High Output). The reverse-stranded paired-end RNA-Seq reads were checked for the presence of adapters and high-quality bases using FastQC (v0.11.7). The reads were later mapped against the Ensembl human reference genome (GRCh38 v109 or GRCm39 v109, based on the respective organism) using the HISAT2 (v2.7.9a; https://daehwankimlab.github.io/hisat2/) mapping tool. The output file from HISAT2 was converted from SAM format to BAM format using SAMtools (v1.9; http://www.htslib.org/). Counts for expressed genes were generated using HTSeq (v0.11.2; https://htseq.readthedocs.io/en/latest/), and output was generated in text format. These count text files were then imported into the Bioconductor R package edgeR (v3.38.4). The edgeR package was then again utilized to identify the differentially expressed genes (DEGs) based on the criteria of the genes having an expression count greater than or equal to the 1.5 fold change (FC) between two experimental conditions and a false discovery rate (FDR) less than 0.05. For both mouse datasets, an exact test was performed to compare 2 groups, while the human dataset used a paired linear model to determine DEGs within the edgeR package. After respective cutoffs of FC > 1.5, *P* < 0.05, and FDR < 0.05, each comparison generated 2,542 DEGs for mouse whole skin, 6,755 DEGs for mouse epidermis, and 2,410 DEGs for human skin. RNA-Seq data are accessible through the NCBI’s Gene Expression Omnibus (GEO) series (accession no. GSE235950). After the DEGs were identified for each experimental comparison, each list of genes along with their differential expression values was uploaded to Ingenuity Pathway Analysis (IPA). IPA results were then compared with selected psoriasis publications using analysis match function in IPA to generate a meta-analysis on a pathway level. *Z* score comparisons and correlation matrix calculated from *z* scores from analysis match were displayed via heatmap and matrix using the pheatmap tool (v1.0.12) in R. We also used BaseSpace Correlation Engine software (formerly NextBio Research, Illumina) to compare our datasets with the published studies on psoriasis and mouse models of psoriasis available through the GEO database, and *P* values for each dataset pair were determined using the Running Fisher algorithm (Illumina) (63).

### LC-MS/MS analysis of lipid mediators.

Lipids were extracted using the Folch procedure (64), and phospholipid phosphorus was determined by a micro-method (65). To prevent oxidation of lipids during extraction and sample preparation, a chloroform-methanol mixture supplemented with 0.01% butylated hydroxytoluene was used. LC-MS analysis of phosphatidylethanolamines (PEs) and their respective oxygenated species was performed on a Thermo Fisher Scientific HPLC system coupled to a Thermo Fisher Scientific Orbitrap Fusion Lumos Tribrid mass spectrometer. Lipids were separated on a normal phase column [Luna 3 μm Silica (2) 100 Å, 150 × 1.0 mm, Phenomenex) as described previously (66). Comprehensive analyses of PE and their oxygenated metabolites were performed with high accuracy by exact masses. The Compound Discoverer software package (Thermo Fisher Scientific) with an in-house–generated analysis workflow and oxidized phospholipid database was used to evaluate LC-MS data. Peaks with a signal/noise ratio greater than 3 were identified and searched against the database of oxidized phospholipids. Lipid signals were further filtered by retention time and *m*/*z* values and confirmed by tandem mass spectrometry (MS/MS) fragmentation analysis. Principal component analysis and orthogonal projection of latent structures–discriminant analysis (OPLS-DA) of PE profiles were performed using SIMCA 18.0 software (Sartorius).

### MALDI-MS imaging.

Frozen normal and psoriatic human skin samples were sectioned (12 μm thick) and applied on conductive indium tin oxide (ITO) glass substrates (Bruker Daltonics). The sections were coated with 1,5-diaminonaphthalene matrix (>98% grade; Fisher Scientific) prepared at 10 mg/mL in 70:30 acetonitrile/water (Optima LCMS grade; Fisher Scientific) using a TM sprayer (HTX Technologies) with a custom method (flow 1.2 mL/min, velocity 1,200 mm/min, 10 psi gas at 3 L/min, spray nozzle 70°C at 40 mm height, “CC” pattern of 9 layers with 2.5 mm spacing). MALDI-MS imaging data were acquired on an Ultraflextreme mass spectrometer (Bruker Daltonics) running FlexImaging 5.0 and FlexControl 3.4 in reflector-negative ion mode (laser focus to minimum, 20 μm imaging locations with no random walking). Samples of control and psoriatic skin were placed on separate ITO slides but analyzed sequentially under the same conditions using the Bruker 2-slide adapter target to control for analytical variables. Monochrome images of PE and oxidized PE (oxPE) abundance localization from the tissues were generated from the MALDI-MS data using SCILS LAB 2023c Pro v11.02.14724 (Bruker Daltonics) for *m*/*z* corresponding to [M-H]^–^ of oxPE species and their non-oxidized PE precursors. These images were saved in TIFF format for import to NIS-Elements v4.30.02 software (Nikon USA). Ratiometric images were produced with modifications to our previous protocol (26). To generate the ratiometric abundance of oxPE to its non-oxidized precursor PE within each 20 μm MALDI-MS imaging location, single channels of oxPE and PE species abundances were extracted with NIS-Elements and combined into a new ND2 file. The color depth was changed to 12-bit (rescaling with oxPE, no rescaling for PE) followed by dividing of the components (oxPE ratio to PE) and scaling by a factor of 5. The resulting monochrome (grayscale) single-channel image was cropped to fit the MALDI analysis region. To localize oxPE abundance with keratinocyte subpopulations, the same tissue section was subsequently stained for immunofluorescence (IF). The resulting IF images were imported into the respective MALDI-MS images, and aligned based on bright-field optical images and fiducial markers.

### Dual secondary ion mass spectrometry imaging.

Dual secondary ion mass spectrometry (SIMS) imaging (Supplemental Figure 5, A–E) was performed on a buncher–ToF SIMS (J105 SIMS, Ionoptika). Details of instrumentation and dual SIMS imaging methods were previously published (27). Fresh-frozen psoriatic and nonlesional human skin tissues were sectioned into 7-μm-thick pieces using a cryomicrotome (Leica CM1850) and placed on gold-coated (200 nm thick) silicon wafers, then stored at –80°C until SIMS imaging.

### Cryogenic (H_2_O)_n_-GCIB-SIMS imaging.

A J105 SIMS water cluster primary ion beam was generated with the high-energy SM GCIB system (Ionoptika). We generated the cluster by passing high-pressure water vapor through a 50 μm aperture; it then underwent supersonic expansion and then collapsed into water clusters. After the ionization process, the water cluster ion beam was mass-selected by a Wien filter and then focused by a double-focus lens with deflectors along the beam column. Size measurement was determined using time-of-flight (ToF) by pulsing through the 0.533 m beam column to the sample surface. The cluster ToF was measured by an oscilloscope (Tektronix TDS2024) with a secondary electron detector. The 70 kV singly charged (H_2_O)*_n_* cluster beam with a ToF of 103 microseconds was selected, equal to cluster size of *n* = 30,900. The beam focus was tested using a 1,000 mesh grid (Agar Scientific) with average beam spot sizes of 3 ± 0.37 for 70 keV (H_2_O)_30k_^+^. A live readout from the data acquisition software (Ionoptika SIMS Mainframe) determined the mass resolution (m/Δm) to be 6,875 at approximately *m*/*z* 100, and 10,000–12,000 up to *m*/*z* 2,000. Skin tissue sections on gold-coated silicon wafers were plunged into liquid ethane and transferred to liquid nitrogen. Under a dry nitrogen atmosphere, the cold sample was inserted to the prechilled cold sample stage in the J105 instrument and maintained at 100 K for the duration of GCIB-SIMS imaging. Guided by the H&E-stained serial sections of the same skin sample, a region of interest was selected for SIMS imaging in negative ion mode using a 70 keV (H_2_O)_30k_^+^ beam. A tiled image was acquired with 128 × 128 pixels within each 400 μm × 400 μm tile at an ion dose of 5 × 10^12^ ions/cm^2^. The metabolites and lipids up to *m*/*z* 2,000 were profiled on each sample. For multiplexed immunostained C_60_-SIMS imaging, an antibody panel and corresponding conjugation kits for linking lanthanide tags to the antibodies were determined (Supplemental Table 21). After they were used for (H_2_O)*_n_*-GCIB-SIMS imaging, frozen skin sections on gold-coated silicon wafers were kept first at –20°C for 1 hour, then at 4°C for 1 hour, followed by fixation in 4% formalin solution at 4°C for 10 minutes and methanol at –20°C for 10 minutes. Tissue sections were blocked with 3% goat serum for 60 minutes at room temperature, stained with an antibody cocktail solution (containing 750 μg/mL of each antibody) at 4°C for 15 hours, washed with 0.2 % Triton X-100 in PBS for 8 minutes, and then stained with nuclear Intercalator-Ir (Fluidigm) at 300 μL/section. After washing with double-distilled water for 10 minutes and air-drying for 30 minutes, slides were again inserted into the SIMS instrument for C_60_ imaging. High-resolution SIMS images using a 40 keV C_60_^+^ primary ion beam were acquired on the same tissue region as was previously done with (H_2_O)*_n_*-GCIB-SIMS. To allow resolution of individual cells, the C_60_^+^ beam was finely focused to 1.16 ± 0.45 μm as measured by line scanning on a scanning electron microscopic image of a 1,000 transmission electron microscopy grid. Stained tissue sections were inserted onto the sample stage at room temperature for positive ion mode imaging. Tiled images were acquired with 256 × 256 pixels within each 256 μm × 256 μm tile at an ion dose of 8.6 × 10^13^ ions/cm^2^. The unique *m*/*z* of the ions specific to each of the isotopic metal tags were mapped to locate the corresponding antibody targets.

### SIMS data processing.

Single mass channels from tiled C_60_ and (H_2_O)*_n_*-GCIB-SIMS images were extracted with Ionoptika Analyze software and used for downstream processing performed with custom-developed Python code. (H_2_O)*_n_*-GCIB-SIMS data were normalized to a stable signal of cholesterol sulfate at *m*/*z* 465.30, which was determined by LC-MS to be unchanged between psoriatic and nonlesional skin samples. The (H_2_O)*_n_*-GCIB-SIMS signal levels of cholesterol sulfate were calculated from 13 randomly chosen regions (15 × 15 pixels each) of the epidermis in psoriatic and nonlesional samples, resulting in a correction factor of 8.6 applied to the psoriasis (H_2_O)*_n_*-GCIB-SIMS data. Coregistration of C_60_ and (H_2_O)*_n_*-GCIB-SIMS images was performed by first selecting mass channels that demonstrated a representative morphology of the tissue and normalizing each to an intensity range of [0, 1]. Normalized images were registered using SimpleITK (v2.0.2) (67) to determine the best affine transformation between C_60_ (fixed) and H_2_O (moving) images by minimizing the mean square difference using a gradient descent optimizer. All H_2_O channel data were then transformed to the C_60_ image space. Nuclear and membrane channels from the C_60_ dataset were used to segment single cells with DeepCell (v0.9.0) (68). Since the (H_2_O)*_n_*-GCIB-SIMS data had been registered to the C_60_ image space, the segmentation instances could be used to extract integrated counts of species in both SIMS datasets. Integrated protein signals from C_60_-SIMS images were used to determine thresholds for cell classifications. Hierarchical clustering analysis on the integrated lipid mass channels from the (H_2_O)*_n_*-GCIB-SIMS dataset was performed with Seaborn (v0.11.1), using the cell types determined from C_60_ images.

### Statistics.

Statistical analysis was performed using Prism v10.0 (GraphPad Software Inc.). Statistical analysis tests utilized in the study include 2-tailed student’s t-test, 1-way and 2-way ANOVA, Pearson correlation, and Fisher’s exact test. A *P* value less than 0.05 was considered significant.

### Study approval.

All studies were conducted in compliance with NIH guidelines for the care and use of laboratory animals and approved by the Institutional Animal Care and Use Committee of the University of Pittsburgh. Human tissues were collected after written informed consent was obtained. All experiments performed with human tissues were approved by the Institutional Review Board of the University of Pittsburgh (20030227 and 19030407) in accordance with an assurance filed with and approved by the US Department of Health and Human Services.

### Data availability.

Raw RNA sequencing data are available in the GEO database under accession number GSE235950. All data supporting the findings of this study are available within the paper and its supplemental material. Values for all data points in graphs are reported in the Supporting Data Values file. Requests for further information and resources and reagents should be directed to and will be fulfilled by the corresponding author.

## Author contributions

KV generated the mouse model. KV, OK, and KS designed and performed the experiments, analyzed the data, and assisted with figure design and manuscript writing. HT and LJS performed SIMS and MALDI-MS experiments, analyzed the data, created the figures, and assisted with manuscript writing. YYT, VAT, and SNS conducted LC-MS experiments, analyzed the data, and assisted with figure design and manuscript writing. A Chang, JW, and URC analyzed RNA-Seq data and assisted with figure design and manuscript writing. JZ, NM, and VKS assisted with experiments and contributed to creating the K14/Gpx4 mice. FG provided resources and assistance with SIMS imaging. ARM assisted with access to human skin samples and provided intellectual expertise. A Chattopadhyay assisted with RNA-Seq data analysis and shared key methodologies. JMP, JAK, and SEW provided patient samples and expertise in cystic fibrosis, acute kidney injury, and asthma, respectively. HB assisted with study design, data analysis, and manuscript writing and provided intellectual expertise. VEK and YLB supervised the project, designed and conducted experiments, provided intellectual expertise, analyzed the data, and wrote the original manuscript. All the authors reviewed and contributed to editing the manuscript before submission.

## Supplementary Material

Supplemental data

Unedited blot and gel images

Supplemental table 1

Supplemental table 2

Supplemental table 3

Supplemental table 4

Supplemental table 5

Supplemental table 6

Supplemental table 7

Supplemental table 8

Supplemental table 9

Supplemental table 10

Supplemental table 11

Supporting data values

## Version 1 11/21/2024

In-Press Preview

## Version 2 01/16/2025

Electronic publication
