# Supplementary material for: Ferroptosis of select skin epithelial cells initiates and maintains chronic systemic immune-mediated psoriatic disease
Source: J Clin Invest. 2024 Nov 21;135(2):e183219. doi: 10.1172/JCI183219 (PMC11735110; doi:10.1172/JCI183219)
Supplement: Supplemental data [file jci-135-183219-s074.pdf]

Supplementary Information

Ferroptosis of select skin epithelial cells initiates and maintains chronic systemic immune-mediated psoriatic disease.

Kavita Vats,[1*] Hua Tian,[2*] Kunal Singh,[1] Yulia Y. Tyurina,[2] Louis J. Sparvero,[2] Vladimir A. Tyurin,[2] Oleg Kruglov,[1] Alexander Chang,[3] Jiefei Wang,[3] Felicia Green,[4] Svetlana N. Samovich,[2] Jiying Zhang,[1] Ansuman Chattopadhyay,[5] Natalie Murray,[1] Vrusha K. Shah,[6] Alicia R. Mathers,[1,7] Uma R. Chandran,[3] Joseph M. Pilewski,[8] John A. Kellum,[9] Sally E. Wenzel,[2] Hülya Bayır,[10,†] Valerian E. Kagan,[2] Yuri L. Bunimovich[1,†]

[1]Department of Dermatology, University of Pittsburgh, Pittsburgh, PA, USA
[2]Center for Free Radical and Antioxidant Health, Department of Environmental Health and Occupational Health, University of Pittsburgh, Pittsburgh, PA, USA
[3]Department of Biomedical Informatics, University of Pittsburgh, Pittsburgh, PA, USA
[4]Biomolecular Imaging Lab, Rosalind Franklin Institute, Didcot, UK
[5]Molecular Biology Information Service, Health Sciences Library System, University of Pittsburgh, Pittsburgh, PA, USA
[6]School of Medicine, University of Pittsburgh, Pittsburgh, PA, USA
[7]Department of Immunology, University of Pittsburgh, Pittsburgh, PA, USA
[8]Department of Medicine, University of Pittsburgh, Pittsburgh, PA, USA
[9]Center for Critical Care Nephrology, Department of Critical Care Medicine, University of Pittsburgh, Pittsburgh, PA, USA.
[10]Department of Pediatrics, Division of Critical Care and Hospital Medicine, Redox Health Center, Vagelos College of Physicians and Surgeons, Columbia University Irving Medical Center, New York, NY, USA
[*]These authors contributed equally.

[†]Corresponding author	[†]Co-corresponding author
Yuri L. Bunimovich, MD, PhD	Hülya Bayır, MD
E1157 Thomas E. Starzl Biomedical Science Tower	3959 Broadway
200 Lothrop Street	New York, NY 10032
Pittsburgh, PA, 15213	hb2753@cumc.columbia.edu
bunimovichyl@upmc.edu	

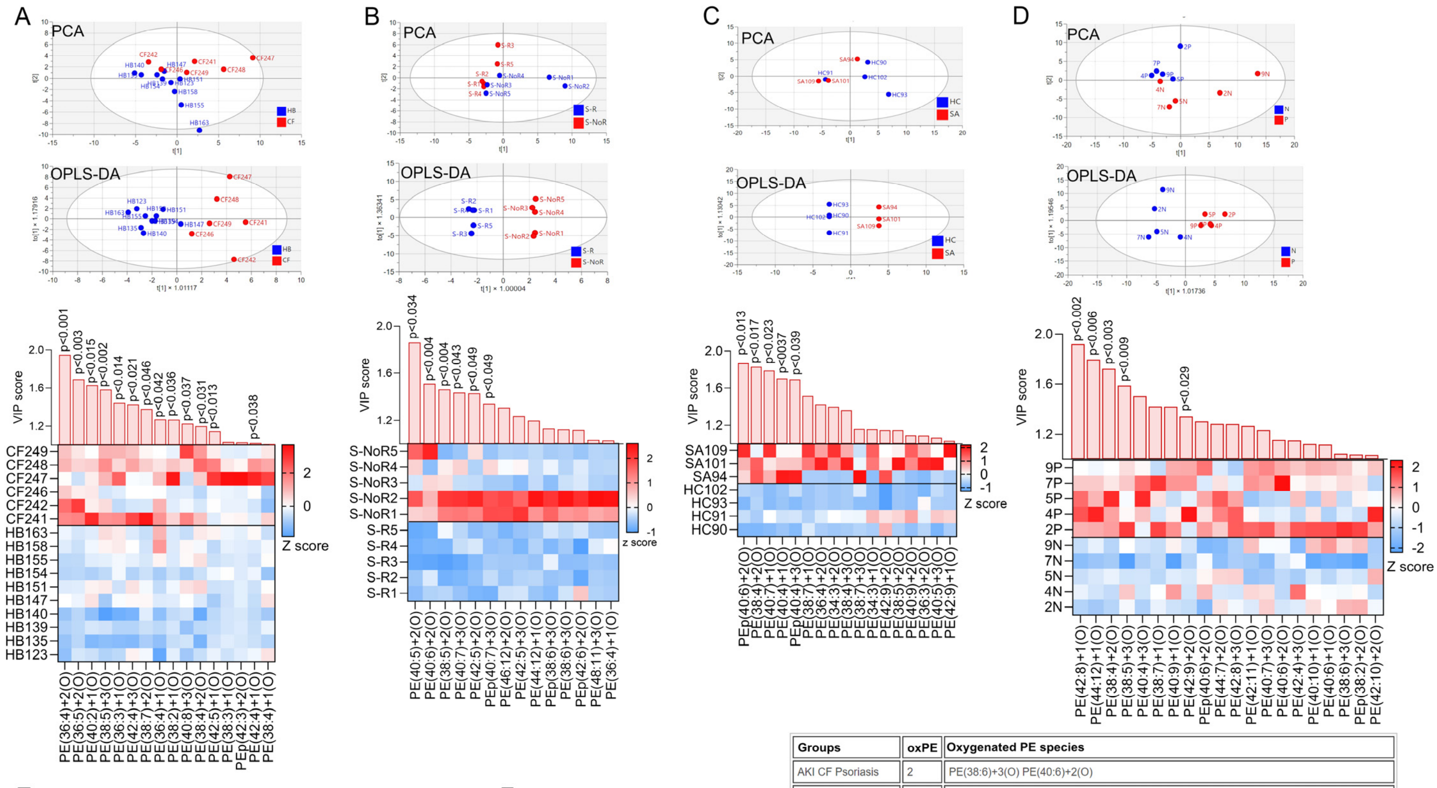

E

ID	VIP score	p value	Δ pmol/ µmol PLs
PE(38:6)+2O	1.983	1.00E-03	-50.40
PE(42:8)+1O	1.919	2.00E-03	10.32
PEp(40:6)+3O	1.905	8.00E-03	-22.21
PE(40:8)+3O	1.831	8.00E-03	-24.78
PE(44:12)+1O	1.795	6.00E-03	25.83
PE(38:4)+2O	1.722	3.00E-03	91.40
PEp(36:5)+2O	1.637	2.00E-03	-7.42
PE(38:5)+1O	1.628	3.40E-02	-31.08
PEp(40:4)+2O	1.503	4.50E-02	-0.58
PE(40:4)+3O	1.502	9.00E-03	3.24
PEp(38:3)+3O	1.377	4.90E-02	-4.62
PE(40:2)+3O	1.356	4.90E-02	-0.75
PE(42:9)+2O	1.341	2.90E-02	2.14

Blue p values: species downregulated in psoriasis.

Red p values: species upregulated in psoriasis

F

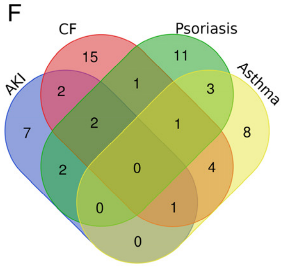

Groups	oxPE	Oxygenated PE species
AKI CF Psoriasis	2	PE(38:6)+3(O) PE(40:6)+2(O)
AKI Asthma CF	1	PE(38:5)+2(O)
Asthma CF Psoriasis	1	PE(38:4)+2(O)
AKI CF	2	PE(36:4)+1(O) PE(40:5)+2(O)
AKI Psoriasis	2	PE(44:12)+1(O) PE(40:7)+3(O)
CF Psoriasis	1	PE(38:5)+3(O)
Asthma CF	4	PE(36:4)+2(O) PE(38:4)+3(O) PE(40:7)+1(O) PE(36:3)+2(O)
Asthma Psoriasis	3	PE(38:7)+1(O) PEp(40:6)+2(O) PE(42:9)+2(O)
AKI	7	PE(48:11)+3(O) PE(46:12)+2(O) PEp(40:7)+3(O) PEp(38:6)+3(O) PE(42:5)+3(O) PEp(42:6)+2(O) PE(42:5)+2(O)
CF	15	PE(38:6)+2(O) PE(38:7)+2(O) PE(46:4)+2(O) PE(40:8)+2(O) PE(36:5)+1(O) PE(38:6)+1(O) PE(42:6)+2(O) PE(40:7)+2(O) PE(36:3)+1(O) PE(40:4)+2(O) PE(40:8)+3(O) PE(38:5)+1(O) PE(38:4)+1(O) PE(40:8)+1(O) PE(34:2)+1(O)
Psoriasis	11	PE(40:4)+3(O) PE(44:7)+2(O) PE(40:10)+1(O) PE(42:11)+1(O) PE(40:9)+1(O) PE(42:8)+3(O) PE(40:6)+1(O) PE(42:4)+3(O) PE(42:10)+2(O) PEp(38:2)+2(O) PE(42:8)+1(O)
Asthma	8	PE(38:7)+3(O) PE(34:3)+1(O) PE(34:3)+2(O) PE(42:9)+1(O) PE(40:5)+3(O) PEp(40:4)+3(O) PE(40:4)+1(O) PE(40:9)+2(O)

Supplementary Fig. S1 (**A**) Airway tissue samples from patients with cystic fibrosis (CF: CF241, CF242, CF246, CF247, CF248, CF249) and from patients without CF (HB123, HB135, HB139, HB140, HB147, HB151, HB154, HB155, HB158, HB163). (**B**) Urine cell pellets from non-recovered (S-NoR) and recovered (S-R) patients after acute kidney injury. (**C**) Pulmonary airway epithelium obtained from patients with severe asthma (SA: SA109, SA101, SA94) and from healthy patients (HC192, HC93, HC91 and HC90). (**D**) Epidermis obtained from psoriatic (lesional) skin (2P, 4P, 5P, 7P, 9P) and from patient-matched perilesional skin (2N, 4N, 5N, 7N, 9N). Contents of oxPE species in each group were used for principal component analyses (PCA), orthogonal partial least squares discriminant analyses (OPLS-DA) with variable influence in projection (VIP) scores (threshold of 1), and heatmaps showing individual oxPE species, quantified as pmol/mg tissue (CF), pmol/nmol of phospholipids (PLs, AKI), pmol/nmol of precursor PL (asthma), and pmol/nmol of PLs (psoriasis), auto-scaled to z-scores and coded blue (low values) to red (high values). Heatmaps show oxPE species with VIP scores >1 which were increased in disease vs. control samples. P-values < 0.05 are indicated over the individual species. (**E**) List of oxPE species up- or down-regulated in psoriatic vs. control epidermis (VIP scores > 1, p < 0.05), derived from OPLS-DA analysis and measured as Δ pmol/µmol of PLs. (**F**) Venn diagram (left) showing numbers of oxPE species (VIP scores > 1, p < 0.05 vs. respective controls) in the epithelial samples of patients with psoriasis, CF, AKI, and asthma. Table (right) indicates oxPE species included in Venn diagram unique to one disease and those identified in two or three diseases.

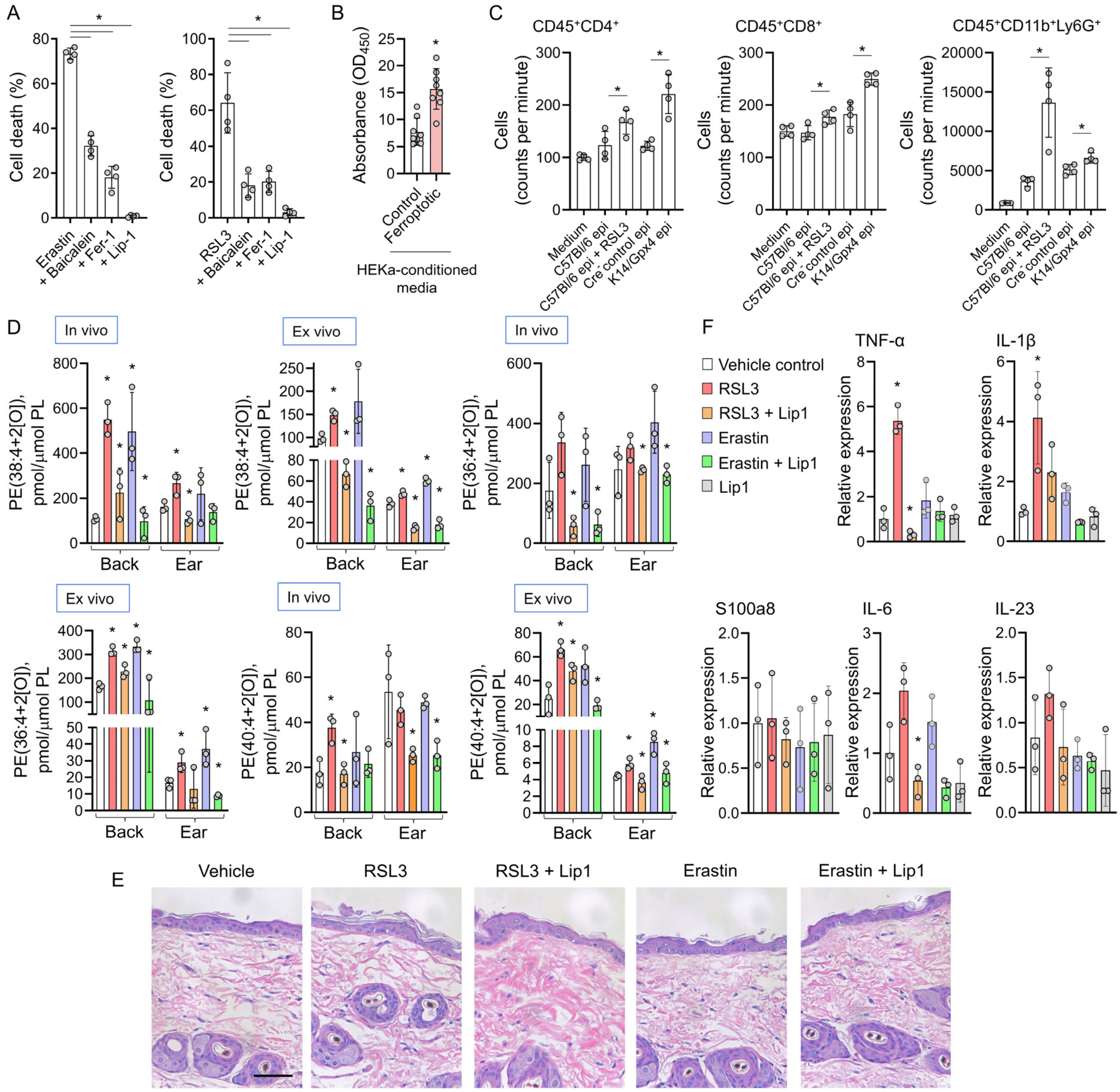

Supplementary Fig. S2 (**A**) Viability of primary human adult epidermal keratinocytes (HEKa) treated for 6 h with erastin (2 µM) or RSL3 (10 µM) alone, or together with baicalein (10 µM), ferrostatin-1 (Fer-1, 1 µM), or liproxstatin-1 (Lip-1, 80 nM). (**B**) ELISA of BrdU incorporation by adult human epidermal keratinocytes (HEKa) cultured in ferroptotic or control HEKa-conditioned media; n=8. (**C**) Transwell migration of C57Bl/6-derived splenic $CD45^+CD4^+$ and $CD45^+CD4^+$ T-cells, and bone marrow-derived $CD45^+CD11b^+Ly6G^+$ neutrophils toward supernatant of epidermis (epi) from C57Bl/6 mice (treated in vitro with RSL3 vs. vehicle control), or toward supernatant of epidermis from K14/Gpx4 or Cre^- control tamoxifen-treated mice. Numbers of trans-migrated cells were determined by flow cytometry and presented as counts per minute; n=4. (**D**) Levels of pro-ferroptotic signals PE(38:4)+2O, PE(36:4)+2O, PE(40:4)+2O (in pmol/µmol of PL) in C57Bl/6 mouse ear or back epidermis treated topically (in vivo) at those locations or harvested and treated ex vivo with RSL3 (10 µM, red bars), RSL3+Fer1 (10 µM, orange bars), erastin (10 µM, blue bars), erastin+Fer1 (green bars) or vehicle control (white bars); n=3 mice per group. Data were normalized to total phospholipids (PL) and presented as pmol/µmol of PL. (**E**) H&E stains of back skin of C57Bl/6 mice topically treated with RSL3 (10 µM), RSL3+Lip1 (2 µM), erastin (10 µM), erastin+Lip1 or vehicle control for 14 days. Scale bar: 50 µm. (**F**) Relative expression of TNF-α, IL-1β, S100a8, IL-6, IL-23 (RT-qPCR) in C57Bl/6 mouse back skin treated for two weeks with RSL3 (10 µM), RSL3+Lip1 (2 µM), erastin (10 µM), erastin+Lip1 or vehicle control; n=3 mice per group. Data are means ± s.d. One-way ANNOVA (A, C, D, F), two-tailed Student's t test (B); *p<0.05 (D, F: RSL3 or erastin vs. vehicle control, RSL3 + [Fer1 or Lip1] vs. RSL3, erastin + [Fer1 or Lip1] vs. erastin).

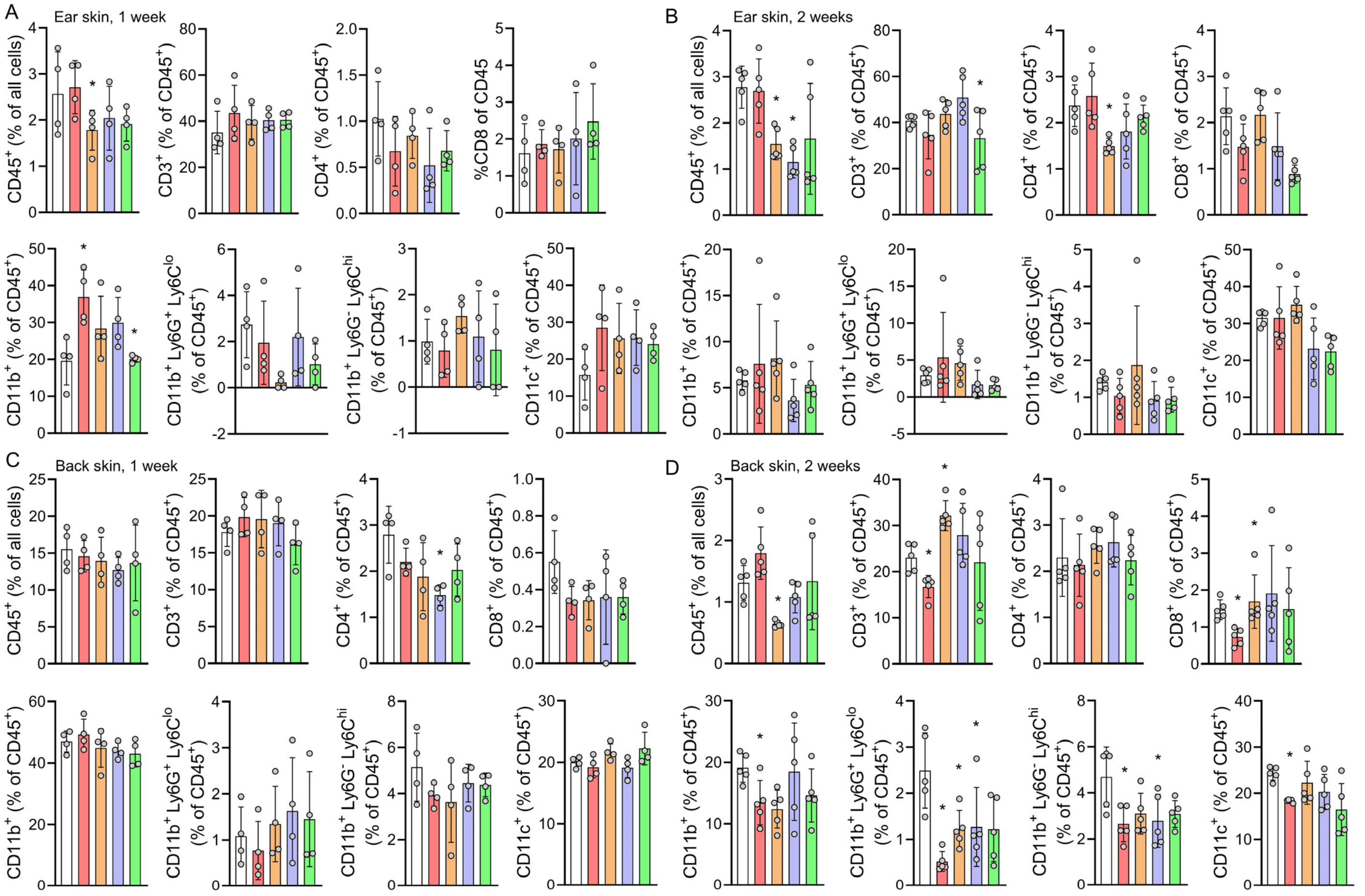

Supplementary Fig. S3 Flow cytometry of immune cell populations in (**A, B**) ear and (**C, D**) back skin of C57Bl/6 mice topically treated with RSL3 (10 µM, red bars), RSL3+Lip1 (2 µM, orange bars), erastin (10 µM, blue bars), erastin+Fer1(green bars) or vehicle control (white bars) for 1 or 2 weeks; n=4 (1 week) or 5 (2 weeks) mice per group. Data are means ± s.d. One-way ANOVA; *$p<0.05$ (RSL3 or erastin vs. vehicle control, RSL3 + Lip1 vs. RSL3, erastin + Lip1 vs. erastin).

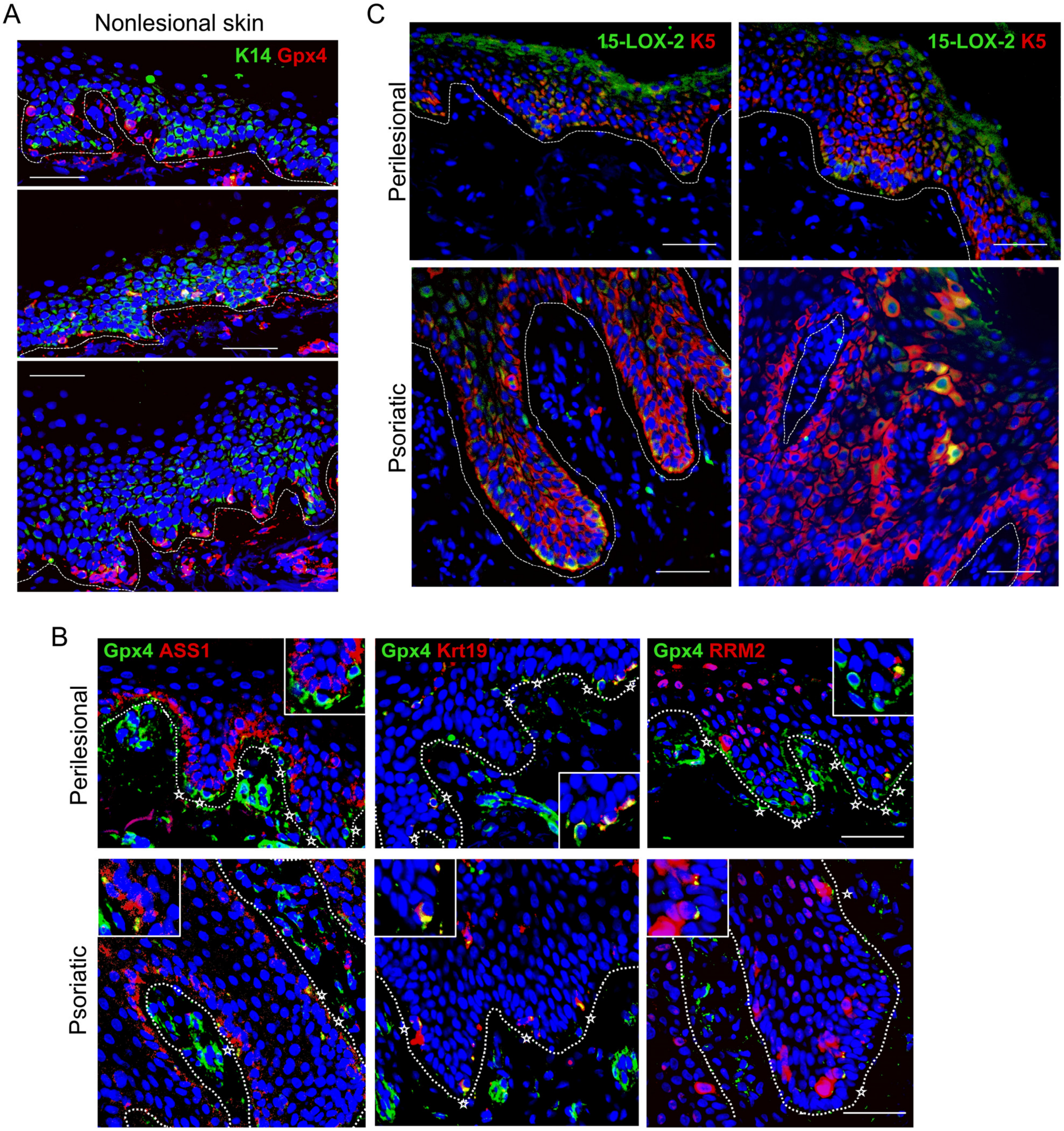

Supplementary Fig. S4 (**A**) IF images of nonlesional human skin. (**B, C**) IF images of psoriatic and perilesional human skin. Insets in (B) show magnified areas from larger images, and white stars identify locations of Gpx4^{+} KCs. Dermal-epidermal junctions are outlined by white dashed lines. Scale bars: 50 μm.

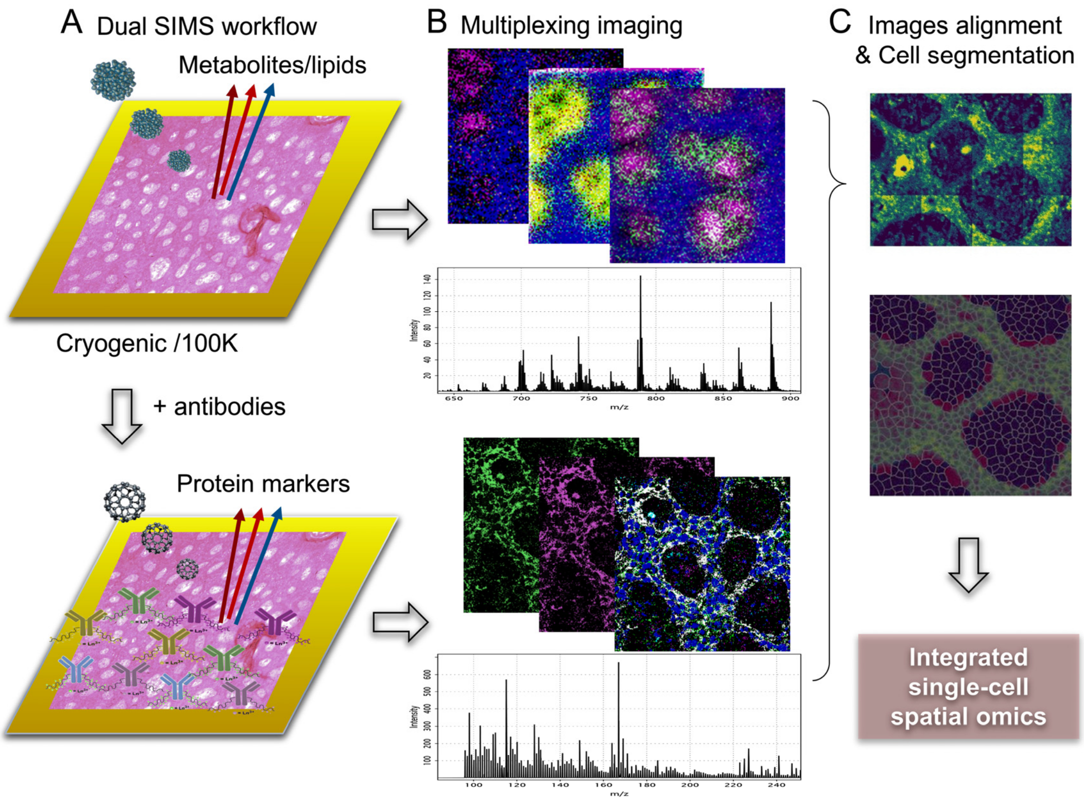

Supplementary Fig. S5 Schematic of integrated single-cell spatial omics of human psoriatic skin using high resolution dual SIMS imaging, $(H_2O)_n$-GCIB SIMS and C_{60}-SIMS. (**A**) First, cryogenic analysis at 100K is performed on frozen-hydrated tissue section for molecular imaging (e.g., lipids and metabolites) using $(H_2O)_n$-GCIB-SIMS at beam spot size of 3 µm. Next, the same frozen tissue section is stained with multiple lanthanide-tagged antibodies for imaging cell-specific markers using C_{60}-SIMS at beam spot size of 1.0 µm. (**B**) Multiplexed imaging of metabolites, lipids and proteins on one tissue section. The $(H_2O)_n$-GCIB generates intact molecular ions up to m/z 2000 with over 160 identified metabolites and lipids with mass accuracy <50ppm. All lanthanide-tagged antibodies are identified by stable isotopic metal ions. (**C**) Lastly, image alignment and cell segmentation are performed to integrate identified metabolites, lipids and proteins at a single-cell level.

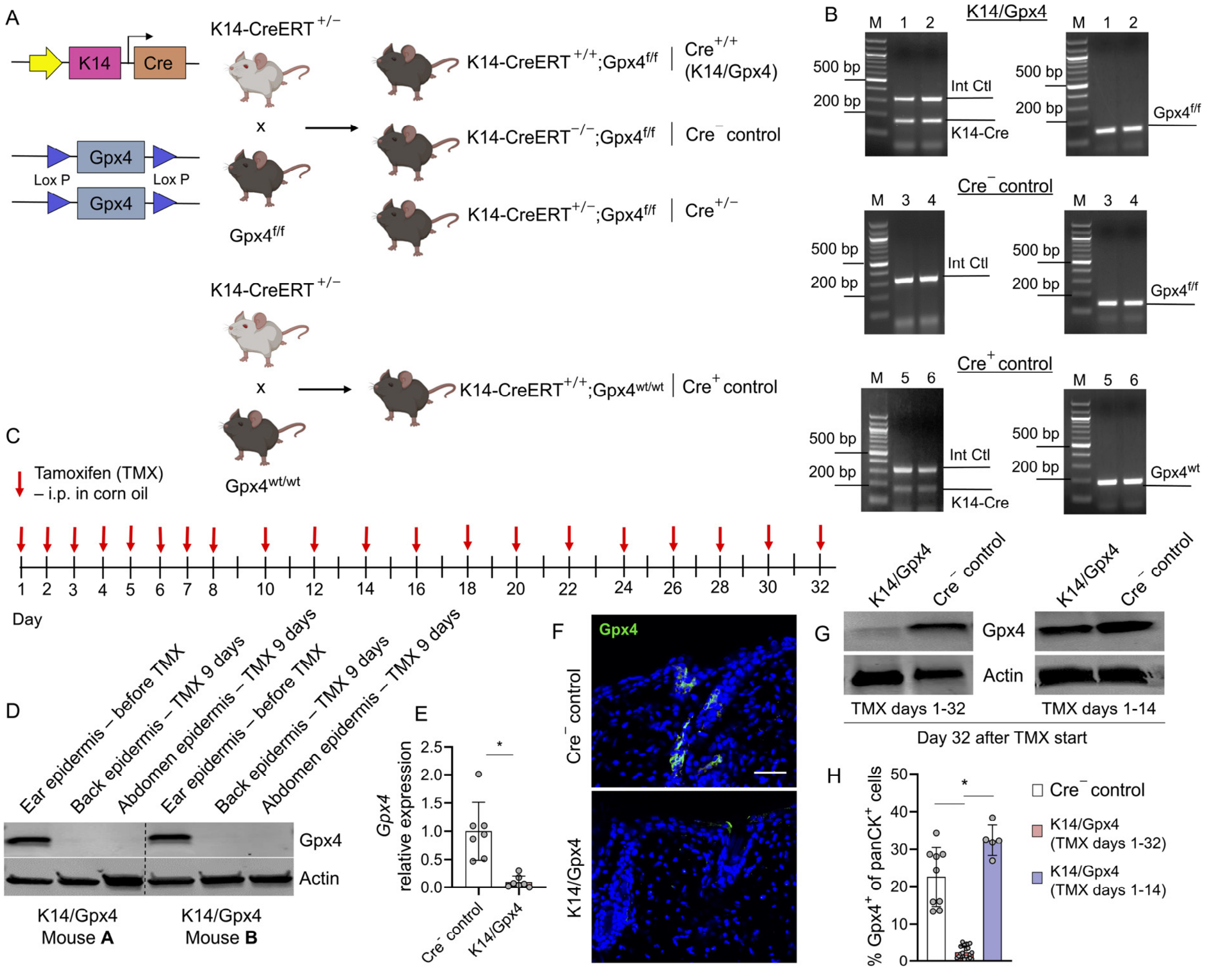

Supplementary Fig. S6 (**A**) Schematic illustrating breeding strategy for the generation of K14/Gpx4 mice, and their littermate controls (Cre$^+$ and Cre$^-$ lines). (**B**) Gel electrophoresis of PCR products derived from F2 generation tail snips of K14/Gpx4 mice, Cre$^+$ controls and Cre$^-$ controls: K14-CreERT and Gpx4$^{f/f}$ transgenes. Lane 1: female F2 pup; Lane 2: Male F2 pup. Internal control (Int Cnt) – 324 bp; K14 transgene (K14-Cre) – 169 bp; Wild-type Gpx4 (Gpx4$^{w/w}$) – 186 bp; Floxed Gpx4 (Gpx4$^{f/f}$) – 142 bp. (**C**) Schematic of tamoxifen (TMX) i.p. injection schedule employed for K14/Gpx4 and control mice. (**D**) Western blot of Gpx4 in epidermis of two K14/Gpx4 mice before and 9 days after TMX initiation. (**E**) Relative expression (RT-qPCR) and (**F**) IF images of Gpx4 in epidermis of K14/Gpx4 vs. Cre$^-$ control mice 28 days after TMX initiation; n=7 mice per group. Scale bar: 50 µm; blue=DAPI. (**G**) Western blot and (**H**) flow cytometry analysis of Gpx4 at day 32 after TMX initiation in epidermis of K14/Gpx4 vs. Cre$^-$ control mice. Each dot represents an individual mouse. TMX was either continued for 32 days or stopped after 14 days. Data are means ± s.d. One-way ANOVA; *$p<0.05$.

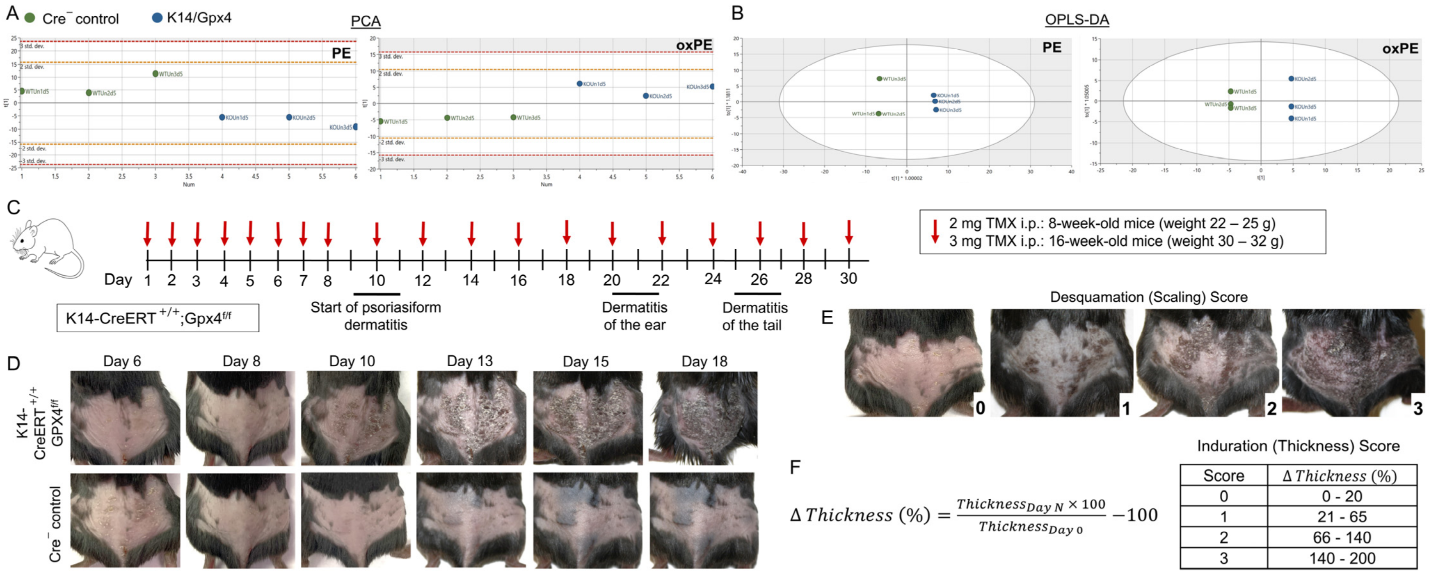

Supplementary Fig. S7 (**A**) Principal component analysis (PCA) and (**B**) orthogonal partial least squares discriminant analysis (OPLS-DA) of K14/Gpx4 vs. Cre$^-$ control mice based on LC-MS measurements of PE and oxPE species in ear epidermis 5 days after TMX initiation. (**C**) Timeline schematic and (**D**) photographs of the emergence of psoriasiform dermatitis in K14-CreERT$^{+/+}$/Gpx4$^{f/f}$ mice relative to the initiation of TMX injections. Illustration of the scoring system (0 to 3) based on (**E**) desquamation (scaling) and (**F**) percent change in the induration (thickness) of back skin of K14/Gpx4 (CreERT$^{+/+}$) mice.

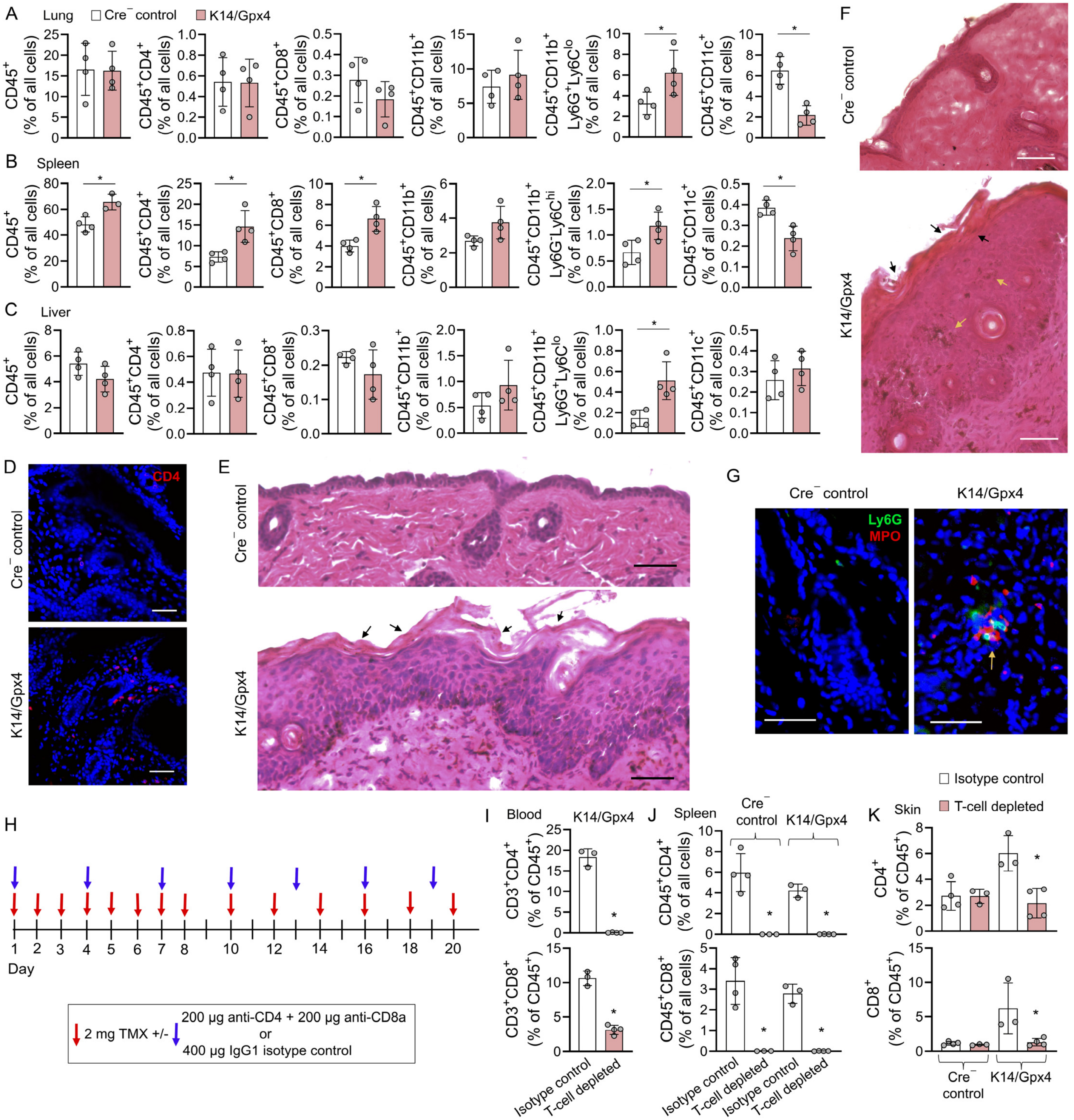

Supplementary Fig. S8 Flow cytometry analyses of immune cell populations in (**A**) lungs, (**B**) spleen and (**C**) liver of K14/Gpx4 and Cre$^-$ control mice 23 days after TMX initiation. Each dot represents an individual mouse. (**D**) IF images of hind paws of K14/Gpx4 and Cre$^-$ control mice 23 days after TMX initiation. Scale bars: 50 μm; blue=DAPI. (**E-F**) H&E images demonstrating psoriasiform features including acanthosis, hypogranulosis, parakeratosis (black arrows) and clusters of intraepidermal neutrophils (yellow arrows) in K14/Gpx4 epidermis vs. Cre$^-$ control. Scale bars: 50 μm. (**G**) IF images showing a cluster of intraepidermal Ly6G$^+$MPO$^+$ neutrophils (yellow arrow) in K14/Gpx4 epidermis vs. Cre$^-$ control. Scale bars: 50 μm; blue=DAPI. (**H**) Schematic of the concurrent administration of TMX and anti-CD4/anti-CD8a or IgG1 isotype control. T-cell depletion in (**I**) blood, (**J**) spleens and (**K**) skin of K14/Gpx4 and Cre$^-$ control mice 20 days after TMX initiation. Each dot represents an individual mouse. Data are means ± s.d. Two-tailed Student's t test (A, B, C, I), one-way ANOVA (J, K); *$p<0.05$.

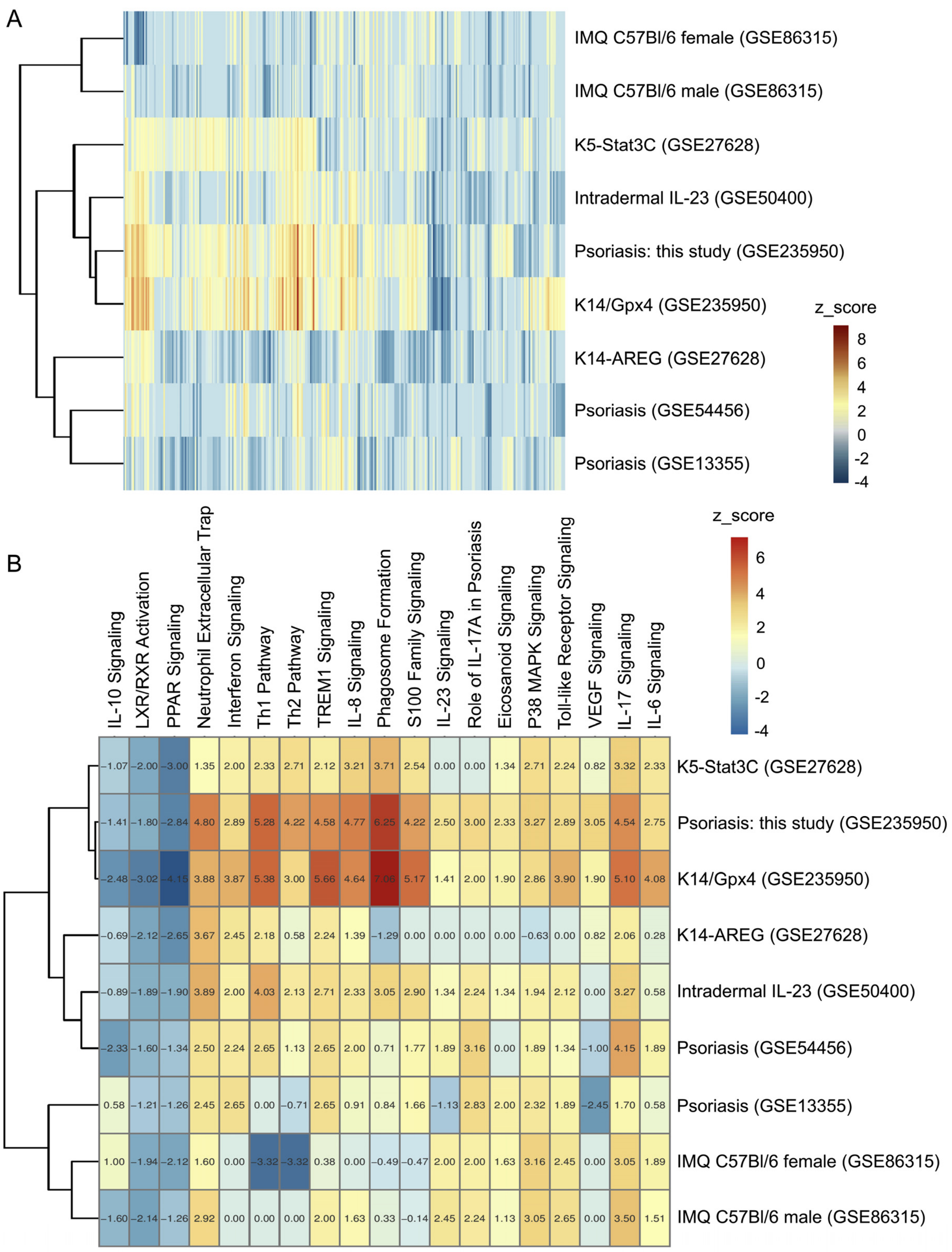

Supplementary Fig. S9 Pathway enrichment analysis (IPA) of 3 psoriasis datasets (including this study) and 5 mouse models (including K14/Gpx4). Clustering based on z-scores of (**A**) all pathways with $p<0.05$ or (**B**) only top 19 psoriasis-associated pathways with $p<0.05$. For any pathway not meeting enrichment cutoff in a dataset, a z-score of 0 was entered.

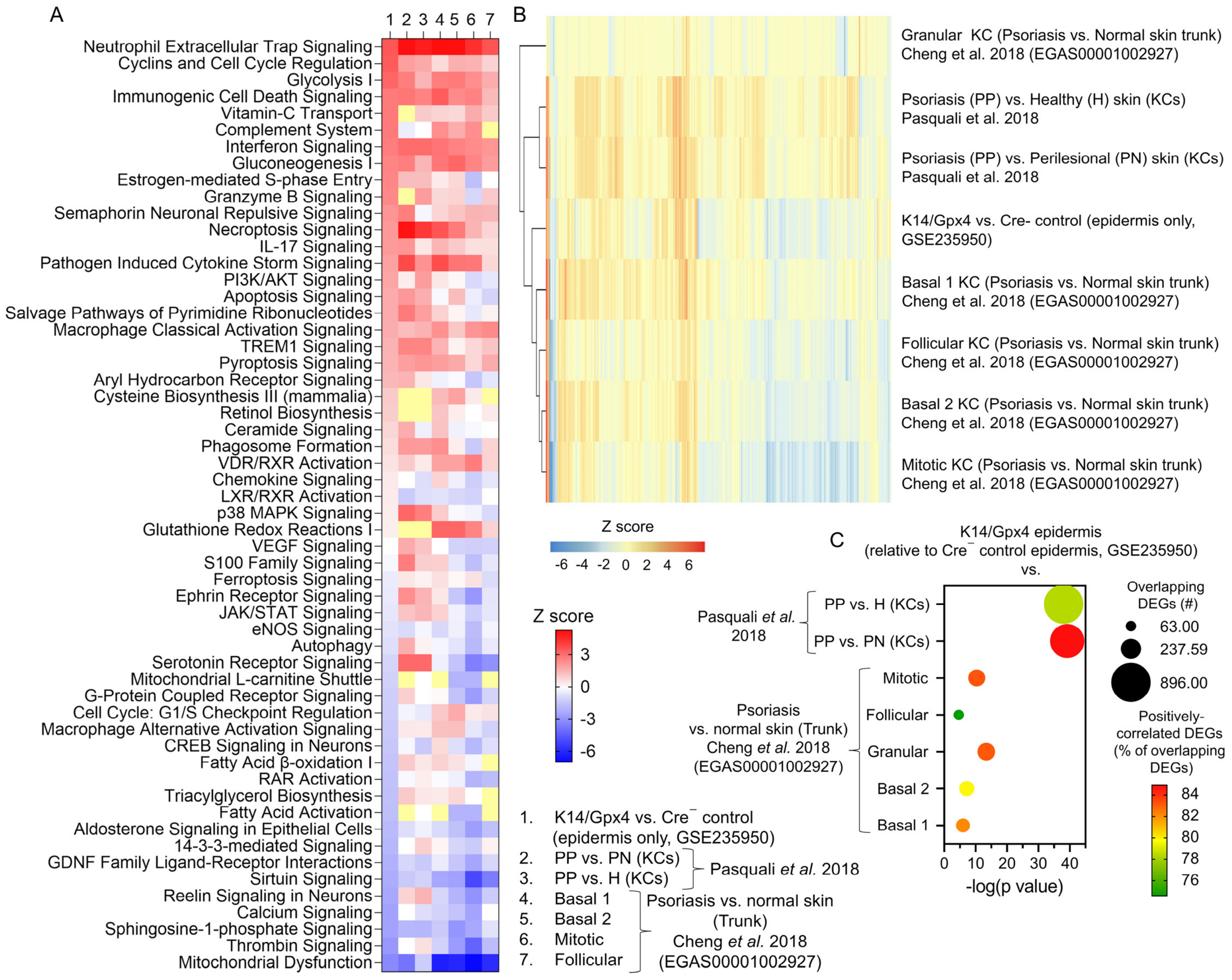

Supplementary Fig. S10 Comparison of enriched pathways (IPA) from K14/Gpx4 epidermis vs. Cre⁻ control epidermis DEG analysis and from two publicly available single-cell transcriptomics datasets of KCs derived from human psoriatic epidermis.[1, 2] (**A**) Select top pathway z scores and (**B**) clustering based on z scores of all pathways with $p<0.05$ are shown. PP=psoriasis, PN=perilesional skin, H=healthy skin. For any pathway not meeting enrichment cutoff in a dataset, a z-score of 0 was entered. (**C**) Correlation analysis of the overlapping DEGs between K14/Gpx4 epidermis (vs. Cre⁻ control epidermis) and KCs isolated from human psoriasis (vs. control normal skin); Fisher's exact test, Illumina Correlation Engine.

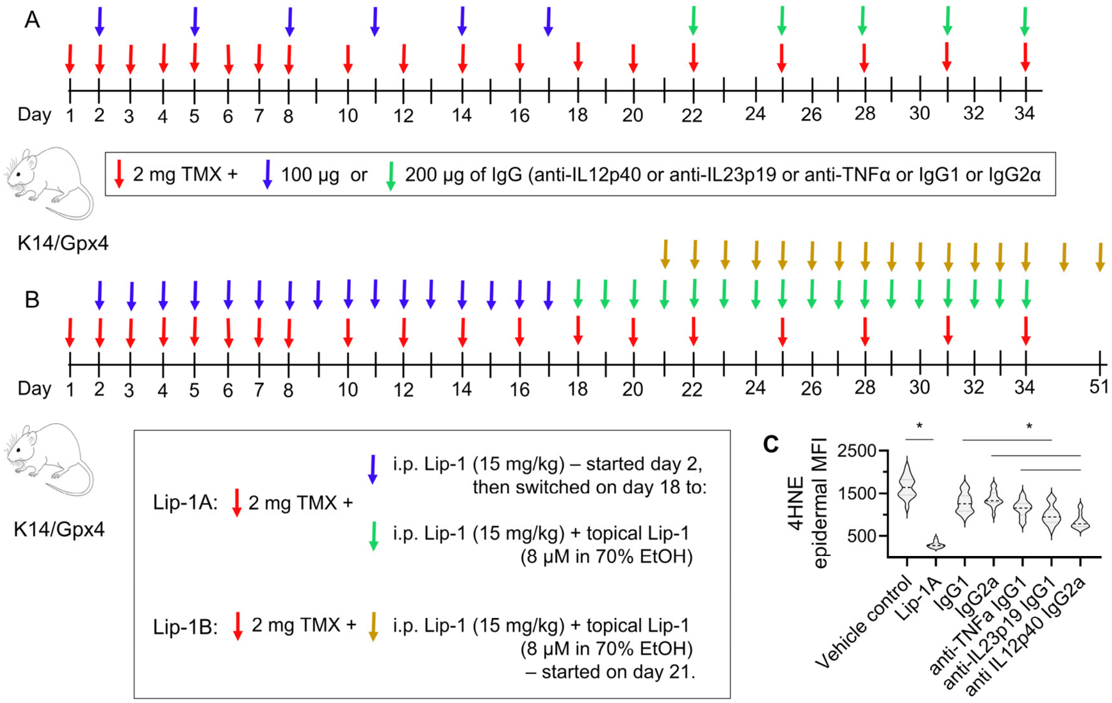

Supplementary Fig. S11 Schematic of the concurrent administration of TMX and (**A**) anti-IL12p40, anti-IL23p19, anti-TNFα antibodies or IgG1/IgG2a isotype control antibodies and (**B**) Lip-1 or vehicle control to K14/Gpx4 mice. In Lip-1A and Lip-1B groups, Lip-1 treatment was started on day 2 and day 21 after TMX initiation, respectively. (**C**) Epidermal MFI of 4HNE in K14/Gpx4 mice treated for 35 days with TMX and vehicle, Lip-1, IgG1, IgG2a, anti-TNFα IgG1, anti-IL23p19 IgG1 or anti-IL12p40IgGa as shown in A and B; n=15 ROIs, 5 mice per group. Data are means ± s.d. One-way ANOVA; *p<0.05.

Supplementary Tables List:

Table S1: Content of PE and oxPE in psoriatic vs. perilesional skin.
Table S2: RNAseq human psoriasis lesional vs. perilesional skin (DEGs).
Table S3: RNAseq human prosiasis lesional vs. perilesional skin (IPA pathways).
Table S4: LC-MS oxPE+2O in mouse epidermis after topical induction of ferroptosis
Table S5: LC-MS PE and oxPE content in K14_Gpx4 vs Cre- control epidermis.
Table S6: RNAseq K14/Gpx4 lesional skin vs. K14-Cre$^-$ control skin (DEGs).
Table S7: RNAseq K14/Gpx4 lesional skin vs. K14-Cre$^-$ control skin (IPA pathways).
Table S8: RNAseq K14/Gpx4 lesional skin vs. K14-Cre$^-$ control skin (GO analysis).
Table S9: Meta analysis K14/Gpx4 vs. human psoriasis and other mouse models (IPA pathways).
Table S10: Meta analysis K14/Gpx4 vs. human psoriasis and other mouse models (Correlation Engine).
Table S11: RNAseq K14/Gpx4 vs. K14-Cre$^-$ epidermis only (DEGs and IPA pathways).
Table S12: Primers used to genotype Krt14-CreERT/Gpx4$^{fl/fl}$ mice.
Table S13: Primers used to differentiate between homozygous and hemizygous Cre.
Table S14: Ferroptosis inducers and inhibitors.
Table S15: Antibodies used *in-vivo*.
Table S16: Antibodies and dye used for flow cytometry.
Table S17: TaqMan probes used for qRT-PCR (Integrated DNA Technologies).
Table S18: SYBR Green aPCR primers.
Table S19: Antibodies used for IF microscopy.
Table S20: Antibodies used for Western blotting.
Table S21: Lanthanide-conjugated antibodies used for SIMS imaging.

Table S12: Primers used to genotype KRT14-CreERT/GPX4$^{fl/fl}$ mice.

Gpx4	Sequence 5' → 3'	Primer type
25814	CTG CAA CAG CTC CGA GTT C	Wild type Forward A
25815	CGG TGC CAA AGA AAG AAA GT	Common
26392	CCA GTA AGC AGT GGG TTC TC	Mutant Forward
		Expected results: Mutant 142 bp, Heterozygote 142 bp and 186 bp, Wild type 186 bp
Krt14	**Sezuence 5' → 3'**	**Primer type**
24964	CGC ATC CCT TTC CAA TTT AC	Transgene Forward
oIMR1374	GGG TCC ATG GTG ATA CAA GG	Transgene Reverse
oIMR7338	CTA GGC CAC AGA ATT GAA AGATCT	Internal Positive Control Forward
oIMR7339	GTA GGT GGA AAT TCT AGC ATCATC C	Internal Positive Control Reverse
		Expected results: Transgene 169 bp, Internal positive control 324 bp

Table S13: Primers used to differentiate between homozygous and hemizygous Krt14-CreER.

Primer	5' Label	Sequence 5' → 3'	3' Label	Primer type	
13593	Fluorophore-1 (FAM)	AAA CAT GCT TCA TCG TCG GTC CGG	Quencher-1	Tg probe	Cre probe
oIMR1084		GCG GTC TGG CAG TAA AAA CTA TC		Transgene Forward	Cre F
oIMR1085		GTG AAA CAG CAT TGC TGT CAC TT		Transgene Reverse	Cre R
oIMR1544		CAC GTG GGC TCC AGC ATT		Internal positive control Forward	
oIMR3580		TCA CCA GTC ATT TCT GCC TTT G		Internal positive control Reverse	
TmoIMR0105	Fluorophore-2 (VIC)	CCA ATG GTC GGG CAC TGC TCA A	Quencher-2	IC probe	

Table S14: Ferroptosis inducers and inhibitors.

Chemical name	Source	Catalog #
Liproxstatin-1 (i.p.)	Selleck	S7699
Liproxstatin-1 (topical, and *in vitro*)	Cayman	17730
Erastin	Sigma	E7781-1MG
(1S,3R)-RSL3	Sigma	SML2234
Baicalein	Tocris	1761
Ferrostatin-1	Sigma	SML0583
Tamoxifen	Sigma	T5648

Table S15: Antibodies used *in-vivo*.

Antibody	Clone	Source	Catalog #
*InVivo*MAb anti-mouse IL-23 (p19)	G23-8	BioXcell	BE0313
*InVivo*MAb anti-mouse IL-12 (p40)	C17.8	BioXcell	BE0051
*InVivo*MAb anti-mouse TNF-α	XT3.11	BioXcell	BE0058
*InVivo*MAb rat IgG2a isotype control	2A3	BioXcell	BE0089
InVivoMAb rat IgG1 isotype control	HRPN	BioXcell	BE0088
In Vivo Plus anti-mouse CD4	GK1.5	BioXcell	BP0003-1
In Vivo Plus anti-mouse CD8α	2.43	BioXcell	BP0061

In Vivo Plus rat IgG1 isotype control	TNP6A7	BioXcell	BP0290

Table S16: Antibodies and dye used for flow cytometry.

Target	Fluorophore	Clone	Source	Catalog #	Dilution
CD45.2	BUV737	104	BD Biosci.	612778	1:100
CD11b	PerCP.Cy5.5	M1/70	BioLegend	101228	1:100
CD3	BUV395	145-2C11	BioLegend	563565	1:100
Ly6C	APC	HK1.4	BioLegend	128016	1:100
Ly6G	BUV395	1A8	BioLegend	563978	1:100
CD4	APC	GK1.5	BioLegend	100412	1:100
CD8a	BV605	53-6.7	BioLegend	100743	1:100
FVD	eFluor 780		eBioscience	65-0865-18	1:1000
CD16/32		2.4G2	BD Pharmigen	553142	1:100
CD11c	BV421	HL3	BD Horizon	562782	1:100
Pan-cytokeratin	PE	C-11	Invitrogen	MA5-28574	1:100
Gpx4	AF647	B-12	Santa Cruz Bio.	Sc-166120	1:100
Cre recombinase		D7L7L	Cell Signaling	15036S	1:100
CD45	BV605	HI30 (RUO)	BD Horizon	564048	1:100
EGFR	BUV737	EGFR.1 (RUO)	BD OptiBuild	748559	1:100
CD49f	PE	eBioGoH3 (GoH3)	Invitrogen	12-0495-82	1:100
CD104	APC	422325	Invitrogen	MA5-23535	1:100
Goat anti-Rabbit IgG	AF488		Life Technologies	A11008	1:100

Table S17: TaqMan probes used for qRT-PCR (Integrated DNA Technologies)

**IL6**	Mm.PT.58.10005566
**IL-1β**	Mm.PT.58.41616450
**IL-17A**	Mm.PT.58.6531092
**IL-23**	Mm.PT.58. 10594618.g
**S100a8**	Mm.PT.58.44003402.gs
**TNF-α**	Mm.PT.58.12575861
**IFN-γ**	Mm.PT.58.41769240
**IL-12p40**	Mm.PT.58.12409997

Table S18: SYBR Green qPCR primers.

Gene	Forward	Reverse
m*Gpx4*	GATGGAGCCCATTCCTGAACC	CCCTGTACTTATCCAGGCAGA
h*Gpx4*	GAGGCAAGACCGAAGTAAACTAC	CCGAACTGGTTACACGGGAA

Table S19: Antibodies used for IF microscopy.

Target	Clone	Source	Catalog #	Dilution
Cre	D7L7L	Cell	15036	1:100
Gpx4		Invitrogen	PA5-102521	1:100
15-LOX-2	D-9	Santa Cruz	271290	1:50
Ki67	SolA15	eBioscience	11-5698-80	1:100
Cytokeratin 14	LL002	Abcam	ab271819	1:100
Cytokeratin 5		Invitrogen	PA5-32465	1:100
CD4 (Alexa 647)	RAM4-5	BioLegend	100533	1:100
Myeloperoxidase (MPO)		R&D	AF3667	1:100
ASS1		Santa Cruz	sc-365475	1:100
RRM2		Santa Cruz	sc-398294	1:100
Cytokeratin 19	OTI3F8	Invitrogen	TA500212S	1:100
4HNE Michael Adducts		Millipore	393207	1:100
Pan-cytokeratin (AlexaFluor 488)	AE1/AE3	Invitrogen	53-9003-82	1:100
**Secondary antibodies**				
Goat anti-rabbit IgG Super clonal Alexa Fluor ™ 647		Invitrogen	A27040	1:5000
Goat anti-rabbit IgG (H+L) Alexa Fluor Plus™ 488		Invitrogen	A32731TR	1:500
Goat anti-mouse IgG (H+L) Super clonal Alexa Fluor™ 555		Invitrogen	A-21422	1:500
Goat anti-mouse IgG (H+L) Super clonal Alexa Fluor™ 647		Invitrogen	A28181	1:500
Streptavidin, Alexa Fluor™ 647 conjugate		Invitrogen	S32357	1:500

Table S20: Antibodies used for Western blotting.

Target	Clone	Source	Catalog #	Dilution
Phospho-Akt (Ser473)	D9E	Cell Signaling	4060	1:1000
Phospho-p44/42 MAPK (Erk1/2) (Thr202/Tyr204)	D13.14.4E	Cell Signaling	4370	1:1000
Phospho-Stat3 (Tyr705)	D3A7	Cell Signaling	9145	1:1000
Akt (pan)	C67E7	Cell Signaling	4691	1:1000
p44/42 MAPK (Erk1/2)	137F5	Cell Signaling	4695	1:1000
Stat3	79D7	Cell Signaling	4904	1:1000
Gpx4	EPNCIR144	Abcam	ab125066	1:1000
GAPDH	D16H11	Cell Signaling	5174	1:1000
Actin	8H10D10	Cell Signaling	3700	1:1000

Table S21: Lanthanide-conjugated antibodies used for SIMS imaging.

Metal	Target	Clone	Catalog #	Source	Conjugation kit
191Ir	Nuclei		201192A	StandardBio	
142Nd	Cytokeratin	AE-1/AE-3	NBP2-23200	Self-conjugated	Maxpar X8 antibody labeling kit (StandardBio #201142A)
148Nd	CK14	LL002	NBP2-34675	Self-conjugated	Maxpar X8 antibody labeling kit (StandardBio #201148A)
174Yb	EGFR	EP38Y	ab272293	Self-conjugated	Maxpar X8 antibody labeling kit (StandardBio #201174A)

References

1. Cheng JB, Sedgewick AJ, Finnegan AI, et al. Transcriptional Programming of Normal and Inflamed Human Epidermis at Single-Cell Resolution. Cell Rep. 2018;25: 871-883.
2. Pasquali L, Srivastava A, Meisgen F, et al. The Keratinocyte Transcriptome in Psoriasis: Pathways Related to Immune Responses, Cell Cycle and Keratinization. Acta Derm Venereol. 2019;99: 196-205.
