# Supplementary material for: Ferroptosis of select skin epithelial cells initiates and maintains chronic systemic immune-mediated psoriatic disease
Source: J Clin Invest. 2024 Nov 21;135(2):e183219. doi: 10.1172/JCI183219 (PMC11735110; doi:10.1172/JCI183219)
Supplement: Unedited blot and gel images [file jci-135-183219-s075.pdf]

Full unedited blots for Figure 4J

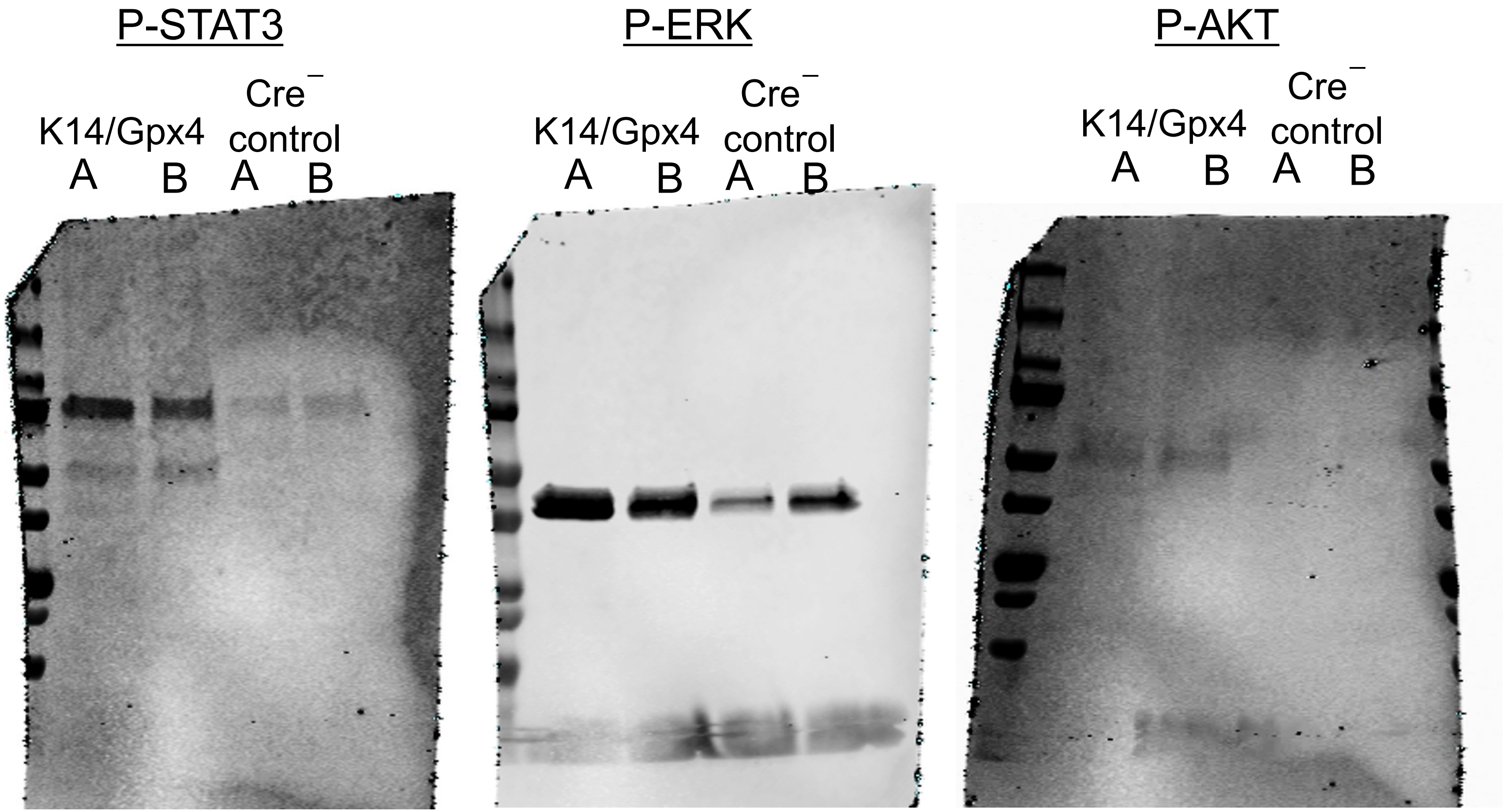

A - male
B - female

Full unedited blots for Figure 4J

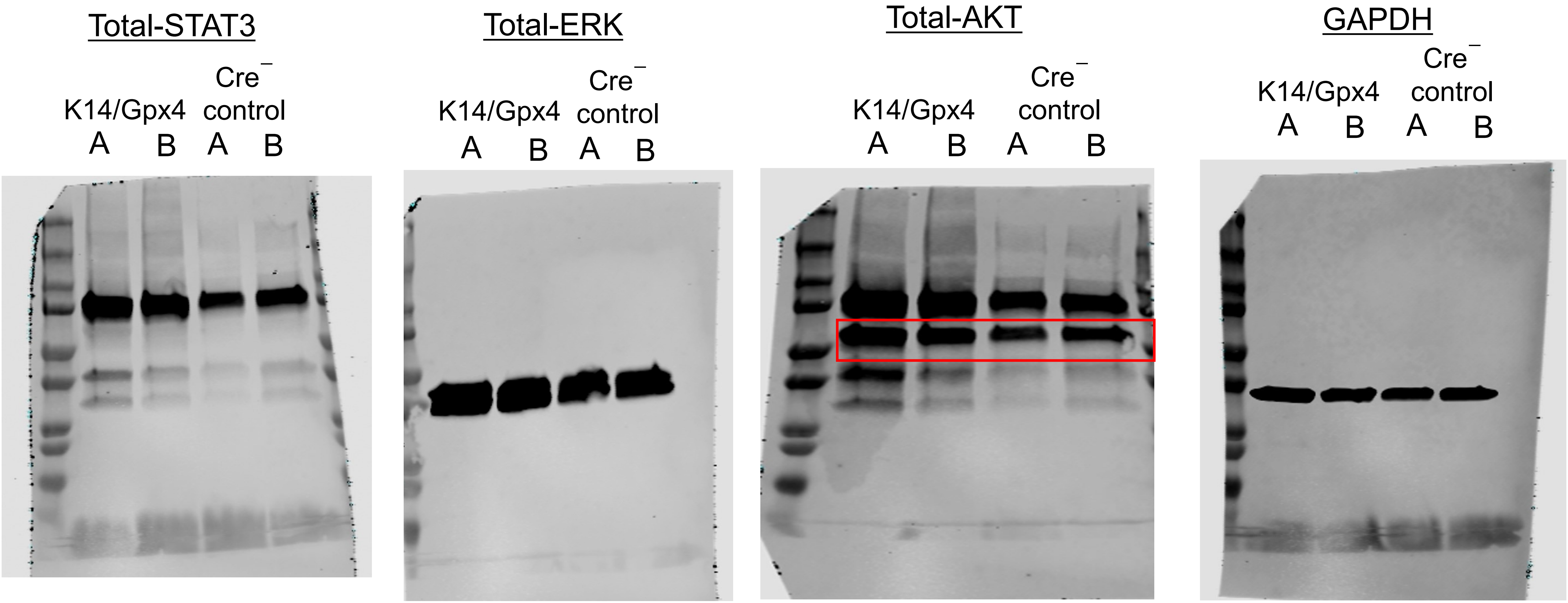

A - male
B - female

Full unedited blots for Supplementary Figure S6D

K14/Gpx4

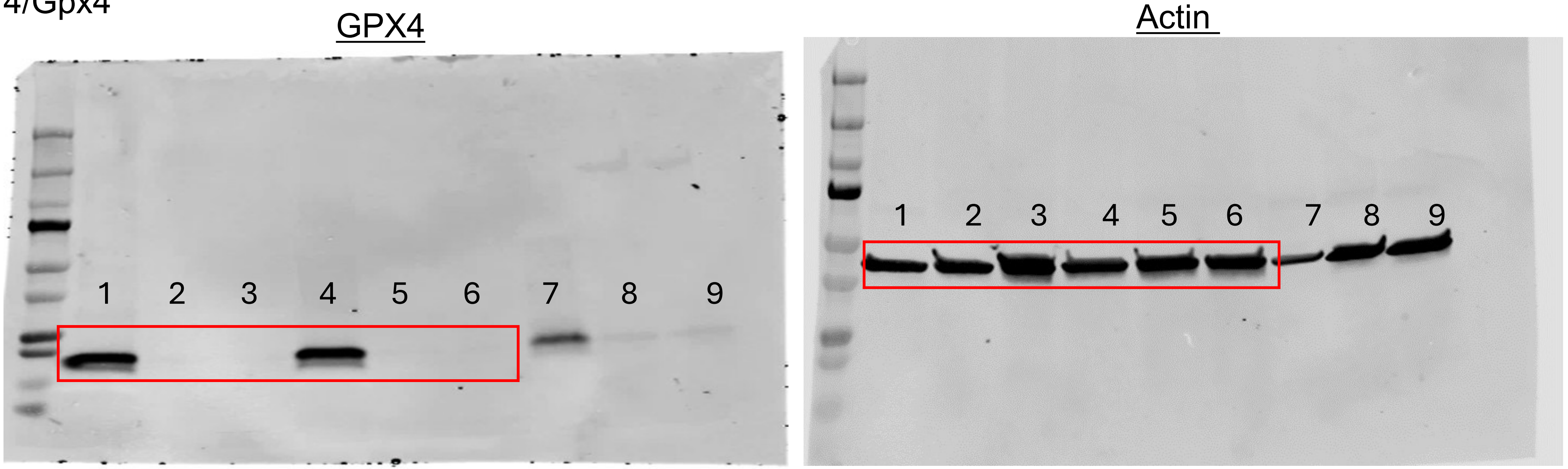

Lane1- K14/GPX4 Ear epidermis before tamoxifen (#194 Mouse A)
Lane 2- K14/GPX4 Back skin epidermis 9 days after tamoxifen (#194 Mouse A)
Lane 3- K14/GPX4 Abdomen epidermis 9 days after tamoxifen (#194 Mouse A)
Lane 4- K14/GPX4 Ear epidermis before tamoxifen (#195 Mouse B)
Lane 5- K14/GPX4 Back skin epidermis 9 days after tamoxifen (#195 Mouse B)
Lane 6- K14/GPX4 Abdomen skin epidermis 9 days after tamoxifen (#195 Mouse B)
Lane 7- Ear epidermis 9 days after tamoxifen Hemizygous Cre (Krt14-CreER$^{+/-}$;Gpx4$^{f/f}$)
Lane 8- K14/GPX4 Back skin dermis 9 days after tamoxifen (#194 Mouse A)
Lane 9- K14/GPX4 Abdomen skin dermis 9 days after tamoxifen (#195 Mouse B)

#194 Mouse A - female
#195 Mouse B - male

Full unedited blots for Supplementary Figure S6G

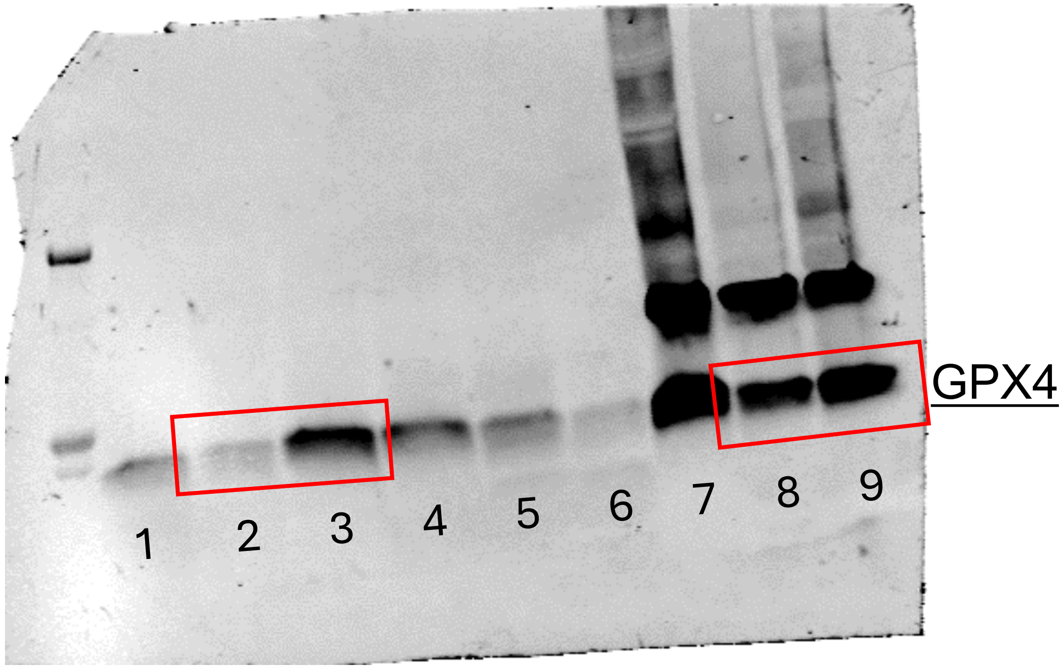

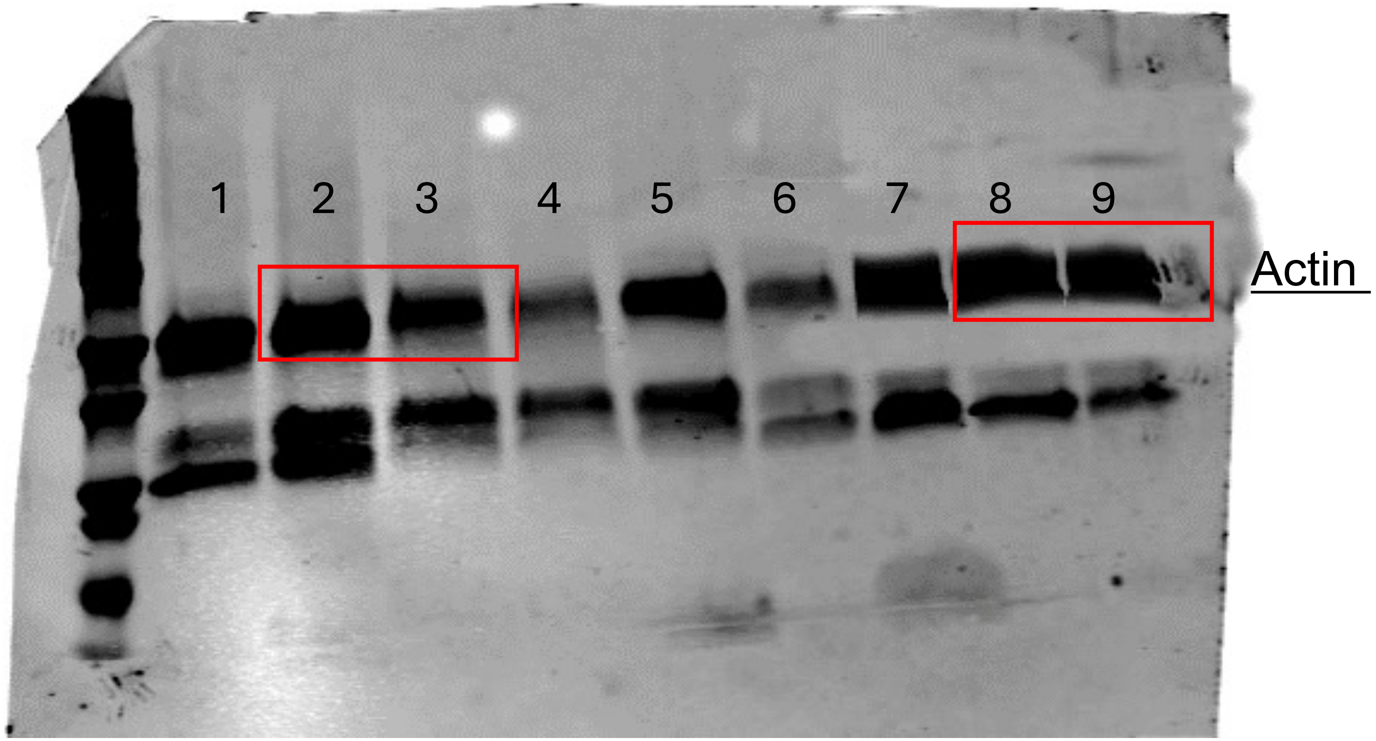

Continuous tamoxifen Exposure (days 1-32)

1- K14/Gpx4 after tamoxifen - male

2- K14/Gpx4 after tamoxifen - female

3- Cre^- control after tamoxifen - female

4- Cre^- control after tamoxifen - male

Tamoxifen stopped on day 14 (days 1-14)

5- K14/Gpx4 after tamoxifen stopped on day 14 – male (collected day 14)

6- K14/Gpx4 after tamoxifen stopped on day 14 – female (collected day 14)

7- K14/Gpx4 after tamoxifen stopped on day 14 – male (collected day 32)

8- K14/Gpx4 after tamoxifen stopped on day 14 – female (collected day 32)

9- Cre^- control after tamoxifen stopped on day 14 – female (collected day 32)
